# A low-cost, portable polarizing digital light microscope for monitoring crystal growth and for crystal harvesting

**DOI:** 10.1107/S1600576726007880

**Published:** 2026-09-04

**Authors:** Sarah Kennedy, Ryan H. Huang, Wojciech Witek, Pawel Krawinski, Coilín Boland, Linjie Yang, Neal Leddy, Martin Caffrey

**Affiliations:** a School of Medicine, Trinity College, Dublin 2, D02 R590, Ireland; b Department of Mechanical and Manufacturing Engineering, School of Engineering, Trinity College, Dublin 2, D02 R590, Ireland; c School of Biochemistry and Immunology, Trinity Biomedical Sciences Institute, Trinity College, Dublin 2, D02 R590, Ireland; Wilfrid Laurier University, Waterloo, Ontario, Canada

**Keywords:** polarizing digital light microscopes, 3D printing, crystal growth monitoring, crystal harvesting, outreach

## Abstract

Motivated by the need for a convenient way to demonstrate the growth of crystals in glass plates by the lipid cubic phase method at crystallization workshops and courses, we developed a lightweight, portable, compact and inexpensive digital microscope device. The device is built around hand-held Wi-Fi and hardwired digital microscopes that are available commercially at low cost and that can use cell phones and personal computers for imaging without the need for unwieldy eyepieces. The device provides illumination from above and below the crystallization plate over a range of brightness levels. It can be used with regular white light and with polarized light to detect birefringent crystals. While not the focus of this study, the new device has been shown to also work with batch and vapour-diffusion plates. Remote monitoring of crystallization plates in a cold cabinet or walk-in refrigerator at 4 °C is possible with the Wi-Fi microscope. Crystals grown in lipid cubic phase and vapour-diffusion crystallization plates were harvested with the aid of the new device and used for diffraction data collection, leading to structure determination at high resolution. Most parts of the device were 3D printed in polylactic acid plastic. The corresponding STL files, included as part of this publication, can be used to make replicates of the current microscope and to produce bespoke devices suited to the specific needs of the user. We envision using the new device for outreach activities at primary, secondary and third-level schools, and at science fairs in support of the citizen scientist. Relatedly, in this study examples are included of recorded images of plant and animal tissue sections. One reveals birefringent materials in a tomato leaf. Another shows optically active crystals of calcium oxalate in onion tunic tissue.

## Abstract 2

A low-cost, portable, medium-resolution polarizing digital light microscope device for monitoring crystal growth and for crystal harvesting is described. The device can be used with a cell phone or a personal computer for real-time imaging by professional and amateur scientists alike.

## 1. Introduction

Crystallography is one of the most powerful methods for establishing molecular structure, thereby providing insights into physical, chemical and biochemical properties and into function. Crystallography has proven indispensable for determining the structures of materials ranging from metals and small organics to complex inorganics and biological macromolecules. Regardless of the material, crystallography relies on the availability of crystals of suitable size and quality. In the area of biological macromolecules, the bulk of crystallography is performed currently with crystals that range in size from single-digit to hundreds of micrometres in maximum dimension (McPherson, 1999 ▸; Rupp, 2010 ▸; Bergfors, 2021 ▸).

Crystals grow from nuclei. Monitoring crystal growth and harvesting crystals for diffraction data collection is typically done with the aid of a compound light microscope. A good quality microscope typically offers single digit micrometre resolution. Microscopes come in a variety of forms with varied functionalities. Typical laboratory microscopes are costly, heavy pieces of equipment with relatively large footprints, they employ unwieldy eyepieces, and they require a power source. Such microscopes are not conveniently available for crystallization demonstrations, courses and workshops or for outreach initiatives when performed outside a crystallography laboratory. Therefore, having available a small, inexpensive, lightweight, portable digital microscope that offers medium to high resolution and polarized light features becomes highly attractive. To satisfy these assorted needs, with a particular focus on the crystallization of membrane proteins by the lipid cubic phase (LCP or *in meso*) method (Caffrey & Cherezov, 2009 ▸; Caffrey, 2021 ▸), the device described in the current report was developed. Hereafter, the instrument will be referred to simply as the digital microscope device (DMD).

The device (Fig. 1 ▸, Fig. S1, Fig. S2) is built around an inexpensive, lightweight, handheld digital microscope (DM), many of which are available via the web for 20 to 30 EUR (30 USD) at the time of writing. The microscope is mounted vertically and attached to a translation slide by means of which the microscopic object, the crystal in a crystallization plate or slide in this case, is brought into focus. This focusing or adjustment axis is referred to hereafter as the *Z* axis. The translation slide is attached to a base in the form of a rectangular prism. The base acts as a stage on which the object sits. It includes a light source for bottom illumination and a rotatable analyser polarizing filter. The base consists of a removable lid, which serves as the mechanical stage on which the crystallization slide sits, and a bottom housing or tray. The slide is moved into viewing position by hand over an aperture in the stage through which light from an LED in the tray passes for bottom illumination. The tray houses a dimmer switch to turn the LED on and off and to control its brightness level. It also includes a disc with a hole in the centre, which is covered by a piece of polarizing filter and a layer of Scotch Magic tape to diffuse the light from the LED below (over which it is centred). Rotating the disc about its centre provides the polarizing feature when operated with a second polarizing filter positioned between the specimen and the objective lens of the microscope. The focusing/working distance of the microscope ranges from 15 to 40 mm, which facilitates convenient crystal harvesting. According to the DM manufacturers, the nominal digital magnification ranges from 50× to 1000× in some models and to 1600× in others (see *Materials* section[Sec sec2.1] below).

In this report, we describe the parts used to build the DMD and where these can be sourced, how to assemble the device, and how to use its features including examining birefringent objects with polarized light. Examples are provided of crystal growth on and harvesting from microscope slides and crystallization trays. Remote monitoring at room temperature (RT, 19–22 °C) of plates in a cold cabinet at 4 °C is also described. Many of the parts used in constructing the microscope were 3D printed. The corresponding static stereolithography (STL) files are included in the supporting information (SI). The device is assembled from readily available and, for the most part, inexpensive parts. With access to a 3D printer, many of the components can be made in the space of a few hours and custom-designed for specific applications. In addition to using the DMD in crystallization and other laboratory settings, we envision placing devices of the type described here in primary, secondary and third-level schools and using them at science fairs for outreach purposes. They should prove useful to the citizen scientist for purposes of inspection, measurement and documentation.

## 2. Materials and methods

### 2.1. Materials

The materials used in building the DMD are detailed in the *Parts List* (SI). All other materials used in this study are listed here. Monoolein (NuChekPrep M239). Chicken egg white lysozyme (UniProt ID, P00698; Sigma L6876, lot 00410643). Sodium chloride (Chemlab, Cat. No. CL00.1423.5000). Sodium acetate pH 4.5 (Hampton Research, Cat. No. HR2-933-10). Glycerol (Fisher Scientific). Acetic acid, PEG 400 (Sigma–Aldrich). PEG 5000 MME (Molecular Dimensions). Cell phone (Samsung, Galaxy A56 5G). PC running Windows 11 (Pro Slim, Dell). Red onion (local grocery store). Standard Reference Material (SRM) 1965 microsphere slide [National Bureau of Standards (NBS), National Institute of Standards and Technology] with calibration 10 µm-diameter polystyrene spheres. Electron microscope 300-mesh grids (TED PELLA Inc.). Light microscope (Nikon Eclipse E400). Microscope slides and cover slips (VWR). Silicon grease (Jena Bioscience, Cat. No. CGR-101). 24-well XRL plate (Hampton Research, Cat. No. MD3-11). Cover slips (glass, 22 mm round, Molecular Dimensions, Cat. No. MD4-04). Water repellent (Rain-X, Stock No. 88197500). Perforated double-stick tape [9500PC 77 mm × 112 mm (6 mm holes), Saunders Inc.]. Glass cutting tool (TCT Scriber and Glass Cutter 150 mm, Tungsten carbide tip, https://www.silverlinetools.com, Cat. No. 633657). Milli-Q water (18.2 MΩ cm, Millipore). Bresser prepared slides (Item number 5984500, https://www.bresser.com). Magnetic wand (Hampton Research, Cat. No. HR4-729). Dual thickness harvesting Microloops (Mitegen).

### 2.2. Methods

#### 2.2.1. 3D modelling and printing

Parts for 3D printing were designed in *Onshape* (https://www.onshape.com/) and *Autodesk Fusion 360* (https://www.autodesk.com/products/fusion-360/overview) (for assembly testing and model refining). The corresponding design files are available as STL files (see SI and Fig. S3, Fig. S4). The STL files were prepared for printing with the open-source PrusaSlicer (https://www.prusa3d.com/). Printing was carried out with an Anycubic Kobra 2 Max 3D printer (suitable 3D printers are often available in schools and public libraries) using polylactic acid (PLA) plastic at 220 °C at a layer height of 0.2 mm and a print speed of 300 mm s^−1^ on a textured polyetherimide plate with 100% cooling. Parts were printed without supports. Burrs, rough surfaces and edges on 3D-printed parts were removed or smoothed using dry aluminium oxide sandpaper (grit size, P280).

#### 2.2.2. Crystallization

Crystals of chicken egg white lysozyme were grown by the *in meso* or LCP method in glass sandwich plates following established procedures (Caffrey & Cherezov, 2009 ▸; Aherne *et al.*, 2012 ▸) that can be viewed in an open-access video (Caffrey & Porter, 2010 ▸). Briefly, 2 parts by volume of an aqueous solution of lysozyme at 50 mg mL^−1^ were combined to homogeneity with 3 parts by volume of the lipid, monoolein, using a coupled-syringe mixing device (Cheng *et al.*, 1998 ▸) at RT. The optically clear, protein-laden mesophase, so formed, was dispensed as a 200 nL bolus into wells created on a glass microscope slide by perforated double-stick tape. One microlitre of precipitant solution [600 m*M* sodium chloride, 100 m*M* sodium acetate buffer, pH 4.5 at RT, 30%(*v*/*v*) PEG 400] was placed on top of the mesophase bolus and the wells were sealed with a glass cover slip. Crystals grew to a maximum dimension of 50–80 µm over a 3–5 h period at RT. Crystals were stable in the mesophase in sealed plates at RT for 2 to 3 days.

The glass sandwich plates were also used to carry out batch crystallization of lysozyme. In this case, a 0.8 µL drop of lysozyme solution (100 mg mL^−1^ chicken egg white lysozyme in 20 m*M* sodium acetate, pH 4.6 at RT) was placed in one half of the well followed by a 0.5 µL drop of precipitant solution [1 *M* sodium chloride, 50 m*M* sodium acetate, pH 4.6 at RT, 30%(*v*/*v*) PEG 5000 MME] in the other half. The precipitant drop was moved slowly into contact with the protein drop to enable the contents of the two drops to mix passively. A glass cover slip was placed immediately on top of the well and tamped into place to seal the well. Plates were incubated at RT or 4 °C. At RT, crystals were obvious in ∼30 min and grew to ∼200 µm in 5 h. Crystals were stable in the sandwich plates for at least 4 weeks at RT.

Crystals were also grown by the hanging-drop (HD) vapour-diffusion method in standard plates following an established protocol (McPherson, 1999 ▸; Bergfors, 2021 ▸). To this end, 2 µL of precipitant solution consisting of 1 *M* sodium chloride and 50 m*M* sodium acetate, pH 4.5 at RT, were combined with 2 µL of a solution consisting of 50 mg of lysozyme per millilitre of milli-Q water on a glass cover slip. The cover slip was inverted and sealed on top of a pre-greased well containing 1 mL of precipitant solution in a 24-well plate at 20 °C. Crystals grew to ∼600 µm in maximum dimension in about 2 days.

#### 2.2.3. Crystal harvesting

Lysozyme crystals were harvested from the mesophase in glass sandwich plates following established procedures that can be viewed open access online (Li *et al.*, 2012 ▸). Briefly, the cover slip sealing the well containing the crystals of interest was scored lightly with a sharp glass cutting tool. A circular section of the cover slip was freed by breaking the glass and was lifted off the well with a pair of tweezers, exposing the mesophase bolus in which the crystals are embedded. The exposed mesophase was covered with a drop (1 µL or less) of precipitant solution to prevent changes in composition and crystal damage. The last harvesting step was carried out on the stage of the DMD using a magnification setting that enabled the entire well to come into view. A harvesting tool/loop (50 to 100 µm, Mitegen) was used to pick out crystals from the mesophase bolus. A minimum of mesophase was taken with the crystals to reduce background X-ray scatter during data collection. The harvested crystals in the loop were immediately snap cooled in liquid nitro­gen without added cryoprotectant and stored in a shipping Dewar for transfer to the synchrotron.

Harvesting crystals of lysozyme from the vapour-diffusion plates involved removing and inverting the cover slip with the hanging drop and placing it on a glass slide on the stage of the DMD. A 500 µm loop (Mitegen) was used to harvest crystals, which were snap cooled in liquid nitro­gen with 50 m*M* sodium acetate pH 4.5, 1 *M* sodium chloride and 20%(*v*/*v*) glycerol as cryoprotectant. Crystals were stored in a shipping Dewar for transfer to the synchrotron.

#### 2.2.4. Macromolecular crystallography, data processing, and structure determination and refinement

Data collection was carried out remotely on beamline I04 at the Diamond Light Source (DLS), UK. Measurements were made in steps of 0.1° at a speed of 20–50° s^−1^ with an EIGER2 XE 16M detector operating in the continuous data collection mode at a sample-to-detector distance of 15–19 cm. Diffraction data were collected with a beam measuring 32 µm × 20 µm, a beam flux of 1.15 × 10^12^ to 1.16 × 10^12^ photons s^−1^ and a wavelength of 0.95373 Å.

Data sets were processed manually using *XDS* (Kabsch, 2010 ▸), and structures were solved by molecular replacement in *Phaser* (McCoy *et al.*, 2007 ▸) using PDB 9kj7 as the search model. The initial model was placed inside a unit cell using *ACHESYM* (Kowiel *et al.*, 2014 ▸) and refined using *Phenix.refine* (Afonine *et al.*, 2012 ▸). Model building between rounds of refinement was performed in *Coot* (Emsley & Cowtan, 2004 ▸). The high-resolution model obtained with the HD crystals was refined with individual anisotropic atomic displacement parameters (ADPs), whereas the moderate-resolution model obtained with the *in meso* crystal was refined using isotropic ADPs and translation–libration–screw (TLS) parameters.

Data collection parameters and refinement and validation statistics are summarized in Table 1 ▸.

#### 2.2.5. Non-crystal specimen preparation

As part of this study, commercially available, prepared slides (Bresser) of onion (*Allium cepa*) root, tomato (*Solanum lycopersicum*) leaf and corn (*Zea mays*) stem were examined with the DMD. The outer skin or tunic of a red onion was also investigated. The sample was obtained by peeling the thin outer layer from the skin of an intact onion. A fragment of tunic was placed on a glass slide along with a drop of water and covered with a cover slip for viewing with the DMD.

#### 2.2.6. Timelapse movie making

Timelapse movies of lysozyme crystals growing by the batch method were made by recording images with the Wi-Fi DMD on a cell phone at discrete time points during the crystallization process. Individual images were copied into *PowerPoint* slides and annotated with elapsed time and a scale bar. The slide deck was converted to a movie in *PowerPoint* with a fade transition between slides of 0.7 s, a dwell time of 2 s per slide and a total playing time of ∼1 min. Movies were saved in *PowerPoint* as MP4 files.

## 3. Results

### 3.1. Components of the DMD

#### 3.1.1. DMD

The DMD introduced here was built around an inexpensive, lightweight, handheld DM (Fig. 1 ▸, Fig. S1) typical of many available commercially via the web. They conveniently run off application software (apps) that can be downloaded to a cell phone or a personal computer. The DM itself includes an objective lens, LED illumination from above the object, a 2D digital CMOS imaging sensor, magnification and illumination brightness adjustment, and a USB power cable. Communication between the microscope and the cell phone or PC can be by means of a hardwire or it can be wireless (hereafter referred to as Wi-Fi). The apps allow for real-time viewing of images on a cell phone or PC screen at different magnifications, and some DMs come with superposed scale bars and calibration rulers. Most provide the option of recording images as static snapshots and as videos at 30 frames s^−1^ for subsequent viewing. In the course of this work, two commercially available DMs were tested, one hardwired and the other Wi-Fi, with maximum nominal digital magnifications of 1600× and 1000×, respectively.

#### 3.1.2. Lighting/illumination

The DMs come with built-in LEDs with which the object under investigation is illuminated from above. There are eight LEDs in total arranged in a ring around the objective lens. Power is provided to the hardwired DM by a USB cable connected to the cell phone or PC. The Wi-Fi DM has a built-in rechargeable battery. Power to the LEDs can be adjusted by means of a switch to turn them on/off and to control brightness levels. The switch is part of the Wi-Fi DM; it is built into the power cable of the hardwired DM.

Bottom illumination on the DMD is provided by a single LED in the base of the instrument (Fig. S2). The LED is powered by one or two replaceable 3 V coin batteries in a battery housing. An off-the-shelf dimmer switch is used to turn the LED on and off and to control its brightness level. The LED comes with wire leads that attach simply to the battery via the battery housing. The battery, in turn, is connected to the dimmer switch by leads on the battery housing. The LED is mounted on a rod with a flat platform at one end to which the LED is attached (Fig. S2A). The LED comes with an adhesive backing which is used to hold it in place on the platform. For a more secure bonding between the LED and the platform, hot glue can be used. The rod can be rotated by hand from outside the base enclosure around its long axis (the instrument’s *X* axis) and repositioned along its long axis to alter the quality of light with which the object is illuminated. This is a useful feature when viewing crystals where facets, edges and corners can be highlighted to varying degrees, thereby providing the viewer with a better appreciation of the crystal’s size, quality and orientation.

The platform on which the LED is mounted has been designed to be long enough to accommodate, in addition to the LED, other materials that can assist in crystal detection and evaluation. When this feature is used, the bottom LED is turned off and slid out of position below the aperture leading to the object so that the end section of the platform is positioned below the aperture. This section can be covered with flat or rumpled tin foil. When used in combination with top illumination from the DM, the reflected light passing at different angles from the foil up through the object can provide a useful and a sensitive way to detect and to evaluate crystals. Instead of tin foil, other materials can be used such as white and coloured paper. Depending on the application, these can provide for a better crystal or specimen viewing experience. The attractive feature of the device is that a variety of materials or none can be tested with little effort or expense.

#### 3.1.3. Polarizing feature

Objects that are optically anisotropic can be conveniently viewed between crossed polarizing filters where they exhibit birefringence which shows up as transmitted light, often of different colours and usually against a dark background. The bulk of the space groups to which crystals of biological macro­molecules belong are anisotropic, and the corresponding crystals can be detected by their birefringence when viewed under polarizing conditions. Accordingly, having a microscope with a polarizing feature is extremely attractive when screening plates for crystal growth. This is especially true for crystals that are grown in the lipid cubic mesophase which itself is non-birefringent. Thus, birefringent crystals show up as bright objects on a dark background, facilitating their detection especially at harvest time. When exposed to air, the surface of the cubic phase can roughen, making crystals in the bolus interior difficult to see under brightfield conditions. However, when viewed with polarized light, the crystals brighten up, becoming visible, and are easily identified for evaluation and for harvesting.

To detect birefringence, the sample is viewed between two polarizing filters with illuminating light passing through all three: the two filters and the sample. One filter is called the polarizer and the other the analyser. The arrangement of the filters with respect to the sample does not matter in the sense that the analyser can be below the sample (the side being illuminated) with the polarizer above or the other way round with the analyser filter on top. To elicit birefringence, one filter (the analyser) must be rotated with respect to the other into the cross-polarized condition where, in theory, no or very little light should get through for detection unless there is birefringent material suitably oriented between the two filters.

In the current DMD, we chose to put the analyser filter in the base of the instrument above the bottom LED and below the sample (Fig. S4). The filter is mounted on a rotatable disc with a small circular aperture in the middle over which the polarizing filter sits (Fig. S2). The filter is held in place on the disc by a strip of Scotch Magic tape, which also serves to diffuse the light from the LED and to provide a more uniform background illumination. The disc rests on ledges designed into three of the side panels of the bottom tray of the base. The aperture in the disc is positioned above the LED and directly below a hole in the lid on which the sample rests. The edges of the disc extend beyond the panels where they can be accessed to rotate the filter by hand.

The polarizing filter, which remains fixed in position and does not rotate during use, is situated between the sample and the objective lens of the DM. The filter is glued at the end of a 5 mm-diameter 3D-printed rod in the form of a paddle (Fig. S4). The rod passes through a hole in the spacer of the DMD mount with the filter positioned just below the objective lens of the DM. When polarization is not required, the plane of the polarizing filter should remain parallel to that of the analyser filter or the polarizer filter can simply be slid out of position below the objective lens. This arrangement means that the polarizing filter can be inserted or removed from the light path with ease by moving the rod in and out of the hole in the spacer.

#### 3.1.4. Assembling the DMD

The sequence of steps in which the DMD is assembled follows.

Step 1. 3D print all the printable parts (Fig. S3). Deburr and smooth surfaces and edges as appropriate.

Step 2. Bring together in one place the 3D-printed parts (Fig. S3) and the other components following the *Parts List* in the SI.

Step 3. Remove the bearing cover from the bottom of the Velmex translation slide. Replace it with the 3D-printed standoff or spacer. Secure the spacer in place with two 3/4′′ 6-32 UNC screws. A 3D-printed alternative to the metal Velmex stage is considered in the *Discussion* section[Sec sec4].

Step 4. Attach the lid of the base to the spacer by means of two M5 screws. Note that the holes into which the M5 screws fit are not threaded. Threading is done by carefully screwing the M5 into the holes. Because of the difference in hardness between the metal screw and the PLA of the spacer, threading is possible. However, it is important not to overtighten the screws in which case the threads can be stripped.

Step 5. Fix the battery holder to the bottom of the tray with hot glue as shown in Fig. S2. Let the glue set for 90 s for a good bond to form. Insert the battery into the holder.

Step 6. Place a bolus of hot glue on the bottom of the base where the dimmer switch is to sit. Insert the knob of the dimmer switch through the aperture in the right-hand panel of the tray. Press the dimmer switch onto the hot glue and hold it in place by hand for 90 s for the glue to set.

Step 7. Insert the LED support rod into its cylindrical housing that is 3D printed as part of the tray. Check that the support rod rotates and translates freely in its housing. If it is too tight, either reprint a new rod of smaller diameter or sand down the existing rod to size. If it is too loose, we have found that a few turns of Scotch Magic tape wrapped around the rod will provide a suitable shim whose thickness can be tailored by the number of wraps to provide a good fit. A good fit is one where, when the rod is translated and/or rotated during use, the rod holds its new position against/remains in place resisting the restoring force and torque imposed by the lead wires connected to the LED.

Step 8. Remove the protective cover from the back of the LED and stick the LED to the flat platform of the support rod. Hold the LED in position for 90 s to provide a secure bond. The exposed platform next to the LED can be covered with tin foil, paper or something else, to provide a reflective surface for viewing samples with top illumination. Hot glue can be used to fix the material to the platform.

Step 9. Attach the leads from the LED to the battery housing and the leads from the battery housing to the dimmer switch as shown in Fig. S2. A Phillips head screwdriver is all that is needed to make these connections. The wire leads attached to the LED and the battery housing can be fixed to the bottom and side wall of the tray using hot glue as in Fig. S2.

Step 10. Tape a small piece of polarizing filter across the hole in the 3D-printed disc using Scotch Magic tape (Fig. S2). Make sure that the filter and the tape both cover the aperture. In addition to sticking the filter to the disc, the tape serves to diffuse the light from the LED, providing a more uniform background for imaging.

Step 11. Place the disc with polarizing filter in place on the ledges in the tray so that the rim of the disc extends beyond the front and two side walls of the tray (Fig. S2). The disc should rotate freely in its seated position.

Step 12. Secure the lid with the translation slide attached to the tray by means of four M4 flat head screws. As in Step 4 above, the holes into which these screws fit are not threaded. Threads can be created by carefully screwing the metal screws into the softer PLA holes at the four corners of the tray. Again, care should be taken not to overtighten the screws to avoid stripping the threads. Be sure the screw heads do not protrude above the surface of the lid as this will interfere with moving sample slides across the stage for viewing. Turn on the bottom LED and, using the rod to which it is attached, centre it by eye below the aperture in the lid. Make a note of the position and orientation of the rod for convenient recentring of the LED during sample interrogation. Turn off the LED.

Step 13. Insert the DM into the holder attached to the translation slide (Fig. 1 ▸, Fig. S4). Orient the DM with the graduated magnification knob facing toward the front of the device for easy access and adjustment. The aperture in the holder is designed to fit the different DM models we have tested during this study. The fit is not exact but is close enough such that the DM can be rocked in position to have it assume, as much as possible, a vertical pose with the objective lens directly over the bottom LED and the illumination aperture in the lid.

Step 14. Place the NBS calibration slide with the 10 µm polystyrene beads on the stage with the beads over the illumination aperture. Adjust the magnification on the DMD to its maximum setting of 1000× or 1600×. Alternatively, the plastic calibration ruler provided with all DMDs can be used in this and the following steps.

Step 15. The DM can be used with a cell phone or a PC. Follow the instructions provided with the DM for downloading the appropriate apps with which to use the microscope. Depending on whether the wired or the Wi-Fi model is being used, the instructions and apps will vary. Since the wired DM offers the higher magnification, we will continue to use it in this assembly demonstration.

Step 16. Plug the wired DM into the cell phone or PC by way of the USB-A or USB-C connector provided. In this example, we will use a PC, which has a larger screen for easier viewing. The software used to interface with the DM is called *HiView*. When *HiView* is launched, a screen appears on the monitor of the PC with images streaming in real time from the detector in the microscope. Turn on the top lighting in the DM. This should register as increased brightness on the screen. Turn off the top lighting and turn on the bottom lighting. The light from the bottom LED should be visible and centred in the image. Assuming the bottom LED is centred below the illumination aperture in the lid, if the light is off-centre on the screen, it can be recentred by tweaking the position of the DM in its holder to the left or right and from front to back. Bring the microscope into focus by adjusting the translation slide. It is convenient to focus on an obvious feature in the sample, such as the round edge of the well, before moving the slide to bring the calibration beads into view. A small adjustment of the translation slide should bring the beads into fine focus (Fig. 2 ▸). We have found that the beads are optimally imaged with the top illumination at full brightness and when the bottom illumination is either turned off or at a very low brightness level. For the most part, the beads appear as hexagonally close-packed arrays. Since each bead is 10 µm in diameter, individual beads or clumps of beads can be used for convenient length-scale calibration. Naturally, the calibration must be repeated with each new magnification setting.

Step 17. The device is now ready for use.

### 3.2. The microscope in use

#### 3.2.1. Resolution and image quality

Since the primary focus of this study was to develop a DMD for use with crystals growing in the lipid cubic mesophase and since crystals of this type are typically in the 10–50 µm size range, a rough estimate of the resolution of the microscope was established using calibration standards. The first of these was the calibration ruler provided by the DM manufacturer. It consists of a thin transparent plastic sheet on which is printed in black a grid and a series of lines and shapes of different sizes. Images of the lines are shown in Fig. 3 ▸. The smallest line is 30 µm wide. It is clearly visible with the DMD under a variety of lighting conditions, suggesting that the resolution of the device is better than 30 µm.

The second calibration standard is an SRM from the National Bureau of Standards, consisting of spherical polystyrene beads that were grown in space under microgravity with a uniform diameter of 10 µm. Images of the beads are shown in Fig. 2 ▸. Individual spheres are distinctly visible, suggesting that the resolution of the microscope extends to at least 10 µm. This resolution is perfectly adequate for screening *in meso* crystallization plates.

The quality of the images that can be viewed in real time and that can be recorded depends on the level of detail in the object under investigation that is sought. In what follows, a selection of images of slides prepared from different biological samples is shown to give the reader a sense of what is possible but also to highlight the limitations of the DMD. The first two slides are sections through a corn stem, an onion root tip and tunic (Fig. 4 ▸), and a tomato leaf (Fig. 5 ▸). In each, the cellular makeup of the tissues is clearly visible, in colour and in reasonable detail. When viewed with polarized light, certain features show up as birefringent (birefringence is examined more fully in a later section), corresponding to calcium oxalate crystals in onion tunic cells (Fig. 4 ▸C.ii) and to starch granules or cellulose or lignin fibres in a tomato leaf (Fig. 5 ▸B). The image of a section through an onion root tip shows the cellular composition of the meristem where the stained nucleus within each cell is clearly visible (Fig. 4 ▸B). Less obvious but still visible is the cell wall, which requires careful adjustment of light intensity and direction (top, bottom, both) and focus to optimize contrast. The take-home message from this sample series is that, provided single-digit micrometre resolution is not needed, the DMD in its current form is perfectly adequate.

#### 3.2.2. Observing and harvesting crystals in the lipid cubic phase in glass sandwich plates

As noted, the DMD was designed primarily as a device for monitoring the growth of crystals in the lipid cubic phase in glass sandwich plates, a procedure that is taught at crystallography and crystallization workshops, schools, and conferences worldwide. The cubic phase method is used pre­dominantly for growing crystals of membrane proteins (Caffrey, 2021 ▸). However, for purposes of demonstrating the method, crystals of more robust and less expensive proteins are generally used. Chicken egg white lysozyme is the test protein of choice, and a protocol was developed whereby crystals of lysozyme grow to maturity in the cubic phase in about 3 h at RT (Aherne *et al.*, 2012 ▸). Accordingly, the new DMD was evaluated as a device for tracking the growth of lysozyme crystals in the lipid cubic phase. The results are shown in Fig. 6 ▸A with the DM set to its magnification maximum of 1600×. Within a couple of hours, small crystals are visible. The cubic phase is itself optically clear. When viewed between two pieces of glass, the crystals appear in an optically clear background. The crystals grow to maturity in 3–4 h, reaching a size of 40–80 µm in maximum dimension. For the most part, the crystals are uniformly shaped as prismatic rods that adopt random orientations at different depths suspended in the highly viscous, 140 µm-thick layer of lipid mesophase in which they grow. Adjusting the focus brings crystals at different depths in the bolus into view.

Crystals in the mesophase can be viewed with top lighting alone, with bottom lighting alone, and with a combination of top and bottom lighting. The user has control over the brightness levels of the top and bottom lighting. It is also possible to translate and to rotate the bottom LED to illuminate the sample and the crystals therein, in slightly different ways. Depending on the lighting options employed, different features of the crystals, such as facets, edges, corners and imperfections, show up to varying degrees. Using reflective tin foil on the platform next to the bottom LED or directly below the microscope slide, in combination with top lighting, can provide a slightly different perspective of the crystals.

The images shown in Fig. 6 ▸A were recorded with fresh crystals that were 5 h old. Accordingly, the crystals are in prime condition, having sharp and well defined facets, edges and corners. Crystals that remain in the lipid cubic phase beyond about 2 days begin to show signs of dissolution, where sharp features are lost and crystals can be seen surrounded by a layer of liquid that likely consists of protein and precipitant solution (Fig. 6 ▸B).

The polarization feature of the new DMD was tested on crystals of lysozyme growing in the lipid cubic phase. The mesophase itself, like the precipitant solution that is used to facilitate crystallogenesis and that bathes the mesophase, is optically isotropic. It is thus non-birefringent and appears dark when viewed between cross polarizing filters. By contrast, the lysozyme crystals in this space group are non-isotropic and exhibit birefringence with polarized light. In Fig. 7 ▸, a number of images of crystals in the cubic phase are shown, recorded with the polarizing filters rotated with respect to one another, ranging from the partially polarizing to the fully polarizing condition. Under fully crossed polarizing conditions and depending on the orientation of the crystal in the bolus, the crystals light up to varying degrees on a dark background. The bigger the crystal the more apparent it is when viewed between crossed polarizers.

Occasionally, the cubic phase will transition to another mesophase state. This can happen if the crystallization well is not closed properly and its contents dry out or a precipitant is used that is incompatible with the cubic phase. Some of these other mesophases are optically non-isotropic and are birefringent. An example is shown in Fig. 8 ▸, where birefringence associated with one of the other phases, the lamellar phase, is apparent and is clearly detected by the DMD.

Harvesting crystals from the cubic phase requires that the plates are opened with a glass cutting tool and that the crystals are retrieved from the viscous mesophase and snap cooled in liquid nitro­gen for shipping to the synchrotron. It is the actual harvesting step that makes use of the new DMD. The *Methods* section[Sec sec2.2] details the procedure, which benefits from practice, patience, a steady hand and good hand–eye coordination. As evidenced by the data in Table 1 ▸, it is possible to recover crystals from the lipid cubic phase and to use them for diffraction data collection. In this case, the crystals yielded a structure to a resolution of 1.74 Å (Fig. 9 ▸).

#### 3.2.3. Observing and harvesting crystals in vapour-diffusion and batch plates

While not the focus of this study, it was interesting to determine if the new DMD could be used with more traditional crystallization plates. This was tested with vapour-diffusion plates in hanging-drop mode at RT. To avoid excessive reflections from the surface of the drops, which can interfere with seeing the crystals, care must be taken to optimally adjust the top and bottom illumination settings. An image is shown in Fig. 10 ▸ where a crystal of lysozyme in a hanging drop is clearly visible. The DMD was subsequently used to assist in the harvesting of crystals from a hanging-drop vapour-diffusion plate. Diffraction measurements made on these crystals yielded a structure with a resolution of 1.07 Å (Table 1 ▸, Fig. 9 ▸). These results show that monitoring crystallization in and harvesting from more traditional vapour-diffusion crystallization plates are both possible with the new DMD.

Batch crystallization of lysozyme at RT in glass sandwich plates was also monitored conveniently by the DMD (Fig. 11 ▸). Snapshots throughout the crystallization process were recorded and converted to a timelapse movie shown in Movie S1. Crystals begin to appear within half an hour of setup and grow to 200–300 µm in about 5 h.

An attempt was made to monitor remotely at RT the batch crystallization of lysozyme in a walk-in refrigerator and a closed cold cabinet at 4 °C using the Wi-Fi DMD. The plates could be viewed remotely provided the cell phone or PC and the DMD in Wi-Fi contact were not physically too far apart. Unfortunately, a complete crystallization screening was not possible under current conditions because the protein came out of solution upon cooling. This was apparent as a band of precipitated protein that spread throughout the protein pole of the crystallization drop. When the plate and DMD were re-equilibrated at RT, the precipitate disappeared and crystals continued to grow, reaching 100–200 µm in a few hours. A timelapse movie of the process is available for viewing as Movie S2.

## 4. Discussion

### 4.1. Image quality

The DMD was designed primarily to quickly track growth of crystals in glass sandwich plates by the *in meso* method as part of a crystallization course, demonstration or workshop. Thus, small size, portability, low weight and affordability were key features incorporated into the original design. The quality of the images, while important, was considered secondary provided the crystals could be easily seen and their quality gauged. That is exactly what this device offers. However, while the images might not be of ‘publication quality’ they can be used to inform the user how the crystallization process is progressing and to show students that the crystallization demonstration has been successful when it is supposed to be and not when the trial is designed to highlight how a crystallization process can go wrong. Both instances can be important in an educational setting. All of this can be realized with the DMD described here. In laboratory situations where a high-quality, expensive professional compound light microscope is not available, the DMD should still enable crystallization screening and optimization trials to continue conveniently and at very low cost. As noted under *Results*[Sec sec3], the DMD can also be employed in the harvesting of crystals, which increases its range of uses and the value of this simple and inexpensive device. The fact that the DMD is low cost and portable means that several can be made available in different parts of a laboratory, including the office (see Fig. 1 ▸), and brought along with the user ‘into the field’ to a synchrotron or free-electron laser facility to assist in crystal selection and possibly harvesting for diffraction data collection.

Note that the digital microscopes used in this study auto-adjust for image contrast and exposure. Although colour images can be recorded, the colour fidelity is not always reliable. Variations in illumination intensity and edge-related optical artefacts can alter the apparent colour of the specimen in recorded images. This effect was observed when imaging histological slides stained with haematoxylin and eosin, such as the colon and oesophagus tissue sections in Fig. S5.

### 4.2. Wi-Fi *versus* wired

Digital microscopes are available commercially in Wi-Fi and wired formats. Apart from the obvious difference in how the two device types connect to or communicate with a cell phone or a PC, among those available commercially the Wi-Fi microscope comes with a maximum nominal digital magnification of 1000× while the wired device offers a digital magnification of 1600×. A considerable portion of the results reported in this study were gathered with a wired DMD. However, we find that the cable attached to the wired DM can cause drag and transmit movement, either of which can affect the position of the microscope in its holder and its centring over the sample. Because the cable may rest next to the focusing knob and because the dimmer switch is built into the cable, the user must handle these (the focusing knob and dimmer switch) with care as even small displacements of the cable can cause significant disturbance of the view at high magnification.

A big advantage of the Wi-Fi DMD is that the device can be set up in one location while the viewing or inspection on a cell phone or PC is carried out at a different location, provided the signal between the two devices is strong enough. Thus, a crystallization trial can be set up, complete with DMD, in a refrigerator at 4 °C while inspection is done on a cell phone or PC in the user’s office or laboratory at more comfortable ambient temperatures (Movie S2). This makes screening at other than ambient temperatures, which is often performed manually (and with much reluctance), more attractive and therefore more likely to be done.

### 4.3. Fluorescence detection

Whilst finding crystals in a crystallization well is a welcome and a positive sign, there is no guarantee the crystal is composed of the desired biological macromolecule or complex. Indeed, the crystal could be composed of salt, lipid, detergent, other buffer components or the partner used for complex formation. To provide a higher degree of certainty that the crystal is of the desired material, in the case of proteins, they can be conveniently labelled with a trace amount of fluorescent dye. If proteinaceous, the crystals should light up when viewed with a fluorescence microscope, which is expensive. The possibility therefore of using the new DMD as a fluorescence microscope becomes very attractive. The dyes commonly used for this purpose have excitation and emission wavelengths in the visible range. Accordingly, it should be possible to replace the LEDs in the microscope of the DMD with LEDs that emit light of a suitable wavelength to excite the fluorescent label of interest, as demonstrated in the work of Tarver & Pusey (2017 ▸). Scattered light from the LED can be filtered out with a low-wavelength filter which, in turn, will permit longer-wavelength fluorescent light from the labelled protein in the crystal to pass through for imaging at the detector. Modifying the DM for this purpose requires opening the microscope and a small amount of soldering to replace the existing LEDs with specially selected LEDs. With these few, reasonably simple modifications, it should be possible to screen for fluorescent crystals in crystallization plates using the DMD. As noted, a proof-of-principle DM of this kind already exists (Tarver & Pusey, 2017 ▸).

A desirable variant on the approach of identifying crystals as being of protein origin is to monitor the intrinsic fluorescence of the protein that derives primarily from its tryptophan residues. The advantage of monitoring a protein based on intrinsic fluorescence, of course, is that labelling with an unnatural dye molecule, which requires additional steps and can be damaging, is not necessary. The challenge, however, is that maximum tryptophan fluorescence occurs with excitation (280 nm maximum) and emission wavelengths (340 nm maximum) in the UV range. While LEDs are available that can be used in this application, the other challenge relates to the fact that standard glass and many plastics absorb UV light. And so, plates and the other optics in the system must be made either of quartz or of a type of glass or plastic that transmits in the UV region. We are investigating these possibilities currently for the purpose of building a DMD that enables UV fluorescence imaging.

### 4.4. 3D-printed translation slide

As much as possible, the DMD described in this report was built using 3D-printed parts. Aside from the digital microscope itself, the other exception to this was the Unislide translation slide. At the time the current project started, the sense was that 3D printing in PLA, or some other plastic, would not produce a stage with threaded and sliding parts having the specifications, in terms of pitch, backlash, reproducibility of positioning, robustness and longevity, available with a metal-based stage. Also, there were several Unislide translation slides in the principal investigator’s laboratory available for scavenging from other pieces of equipment no longer serviceable. In the spirit of sustainable waste management and resource conservation, one was used to build the current DMD. However, at time of writing, these stages cost 220 USD. We are currently exploring the possibility of 3D printing a translation slide using the latest methods and materials which provide a device with the desired performance specifications at a considerably reduced total cost.

### 4.5. 3D prints as STLs

An attractive feature of the current DMD is that many of its components are 3D printed and all the corresponding STLs, upon which the printing is based, are available as part of the SI. These can be used to generate more of the same parts or modified with ease to suit the specific needs of users. Thus, for example, the current DMD was designed for use with *in meso* crystallization plates prepared from standard 1′′ by 3′′ glass microscope slides. With larger 96-well glass plates or with more traditional vapour-diffusion 24- or 96-well plates, a bigger base can readily be designed and printed. The polarizing disc and the slots in the tray walls on which the disc sits will also have to be enlarged.

The current DMD was designed around two commercially available digital microscopes. It is possible that, in time, new and improved variants will become available. Some of the specifications for these new microscopes, such as the outer diameter or shape of the microscope tube itself, may also change. These can be accommodated in a revised DMD simply by adjusting the corresponding STL files in the SI.

Other modifications that can be implemented include making a holder for the upper polarizing filter that sits between the specimen and the objective lens in the digital microscope. This could be a 3D-printed part that sits on the snout of the microscope directly below the objective lens. It should, however, be designed for easy installation and removal while the microscope is in use. The current design, where the filter is part of a paddle-shaped insert, offers this attractive feature of easy insertion and removal.

To facilitate harvesting, it is useful to secure the glass slide to the stage by some means other than one of the user’s hands. Both hands are usually required for the actual harvesting operation. One hand is typically employed manipulating the harvesting loop while the other steadies the hand doing the actual harvesting. That other hand might also be required to adjust the focus and/or magnification during harvesting. To secure the slide in place and free up one of the user’s hands, we have used capillarity and surface tension by wetting, with a couple of drops of water, the surface of the stage on which the slide sits. Clamping clips could also be installed. These are available on the web at very low cost. However, their use would require slight modification of the lid with two holes in which to place the pins that help secure the clips.

### 4.6. Threaded inserts for strength

The current DMD was designed with a focus on simplicity. However, simplicity can come at a cost. Relatedly, we have taken a shortcut for the purpose of securing parts of the DMD together by using metal screws that are screwed directly into 3D-printed PLA. Because PLA is a much softer material than most metals, if the metal screw is overtightened, the threads in the PLA printed part will strip. This is not catastrophic as the part can be reprinted. However, in the interests of strength and durability, it is possible to use threaded inserts into which the screws can be fastened. The inserts are made of metal and are secured in holes in the PLA part where the screw inserts. Insertion is facilitated by heating the threaded insert to melt the PLA while the insert is forced into the receiving hole. On cooling, the PLA solidifies around the insert, fixing it in place. This provides a metal thread into which a metal screw can be fastened. There are YouTube videos available that show how the insertion process is done (see this video for example: https://youtu.be/G-UF4tv3Hvc).

## 5. Conclusions

A low-cost, lightweight, portable digital microscope device is described that can be used to monitor the growth of biological macromolecular crystals in crystallization plates. The device offers spatial resolution to at least 10 µm and incorporates polarizing filters for tracking the growth of birefringent crystals. Crystal growth can be monitored in real time on a cell phone or a PC and recorded as snapshots or videos. The device can also be employed to assist in the harvesting of crystals from different plate types for use in high-resolution structure determination, as demonstrated in this study. Monitoring crystallization in a walk-in refrigerator or cold room remotely for user-friendly operation at ambient temperatures is possible with the Wi-Fi version of the device. The digital microscope upon which the device is based is commercially available at very low cost. Many of the parts in the device can be 3D printed in plastic using the STL files included with this report. The STL files can be modified easily to build bespoke variations on the existing instrument. Because of its many attractive features, we envision the device being used in a range of laboratory types, in offices, and in outreach activities at courses, at workshops, in schools, at science fairs and in the field for the benefit of the citizen scientist.

## Supplementary Material

Supporting information file. DOI: 10.1107/S1600576726007880/dv5032sup1.pdf

Movie 1. DOI: 10.1107/S1600576726007880/dv5032sup2.mp4

Movie 2. DOI: 10.1107/S1600576726007880/dv5032sup3.mp4

Disc STL. DOI: 10.1107/S1600576726007880/dv5032sup4.stl

LED Support Rod STL. DOI: 10.1107/S1600576726007880/dv5032sup5.stl

Lid STL. DOI: 10.1107/S1600576726007880/dv5032sup6.stl

Paddle for Polarizing Filter STL. DOI: 10.1107/S1600576726007880/dv5032sup7.stl

DM Holder STL. DOI: 10.1107/S1600576726007880/dv5032sup8.stl

Spacer STL. DOI: 10.1107/S1600576726007880/dv5032sup9.stl

Tray STL. DOI: 10.1107/S1600576726007880/dv5032sup10.stl

PDB reference: lysozyme *in meso*, 30db

PDB reference: lysozyme hanging drop, 30da

## Figures and Tables

**Figure 1:**
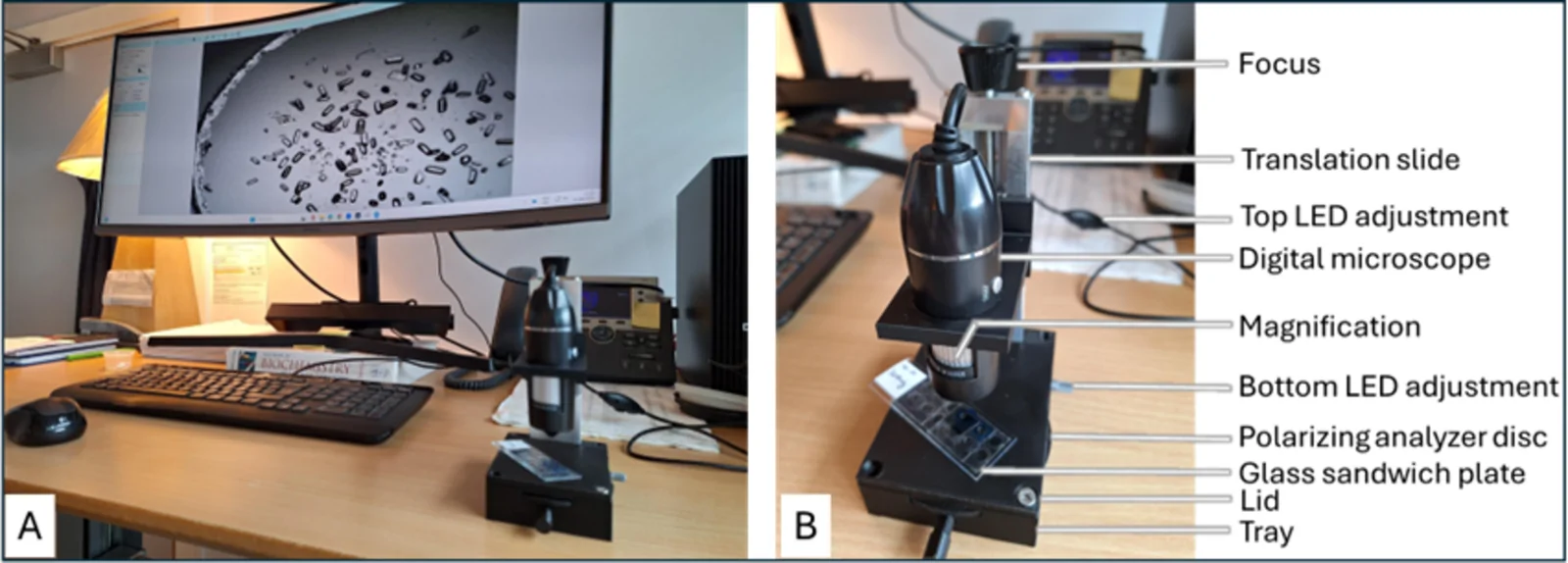
The digital microscope device (DMD) in use. (A) Wired DMD in use in the principal investigator’s office on a PC. Lysozyme crystals growing in the lipid cubic phase in a glass sandwich plate are imaged with the *HiView* app. (B) Close-up view of the DMD with parts labelled.

**Figure 2:**
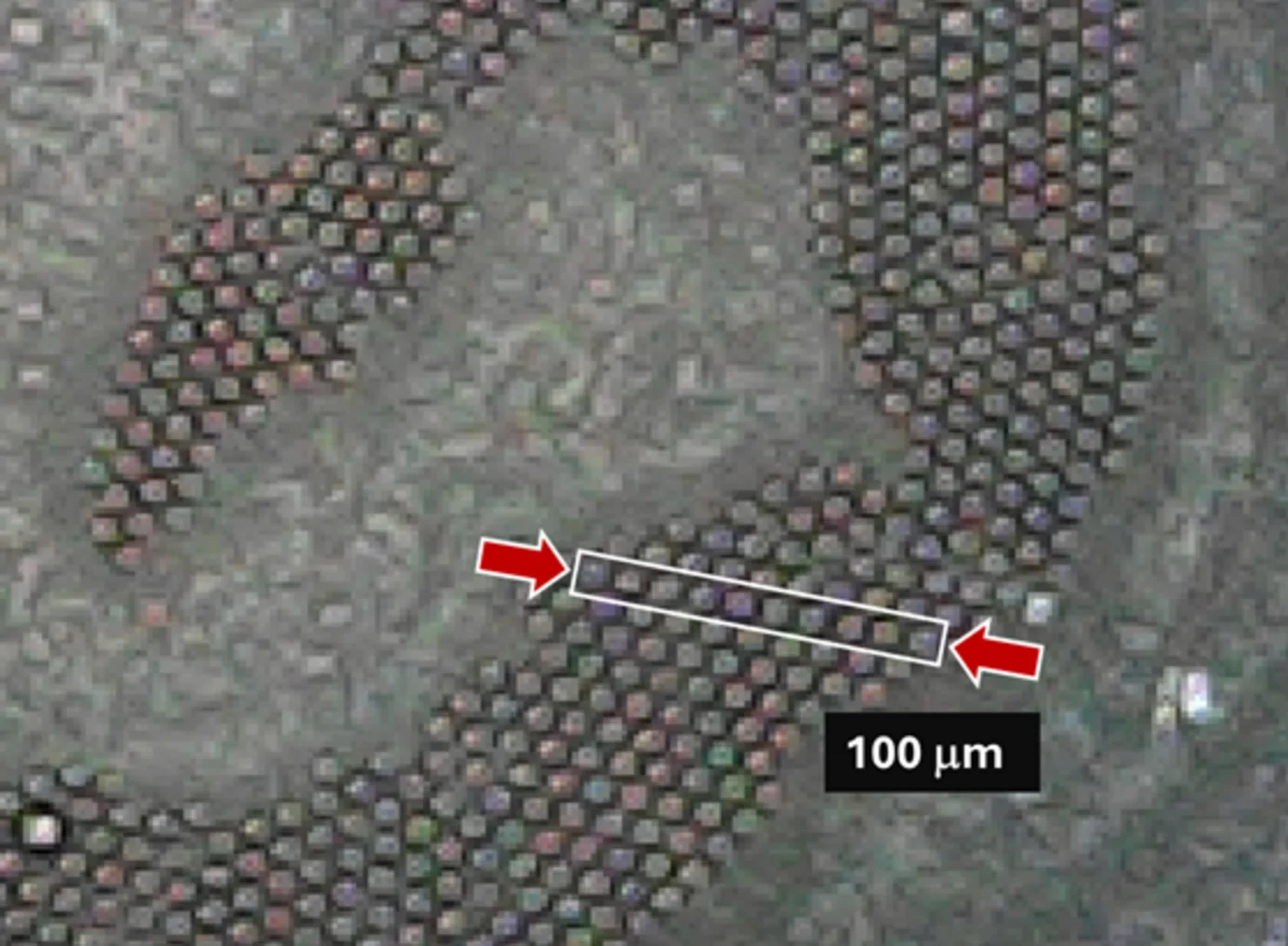
SRM spherical polystyrene beads on a prepared glass slide (SRM 1965 Microsphere Slide) imaged with the wired DMD. The beads were grown in space under conditions of microgravity and have a mean diameter of 9.89 ± 0.04 µm. The distance between the two arrows that bracket 10 beads corresponds to 100 µm.

**Figure 3:**
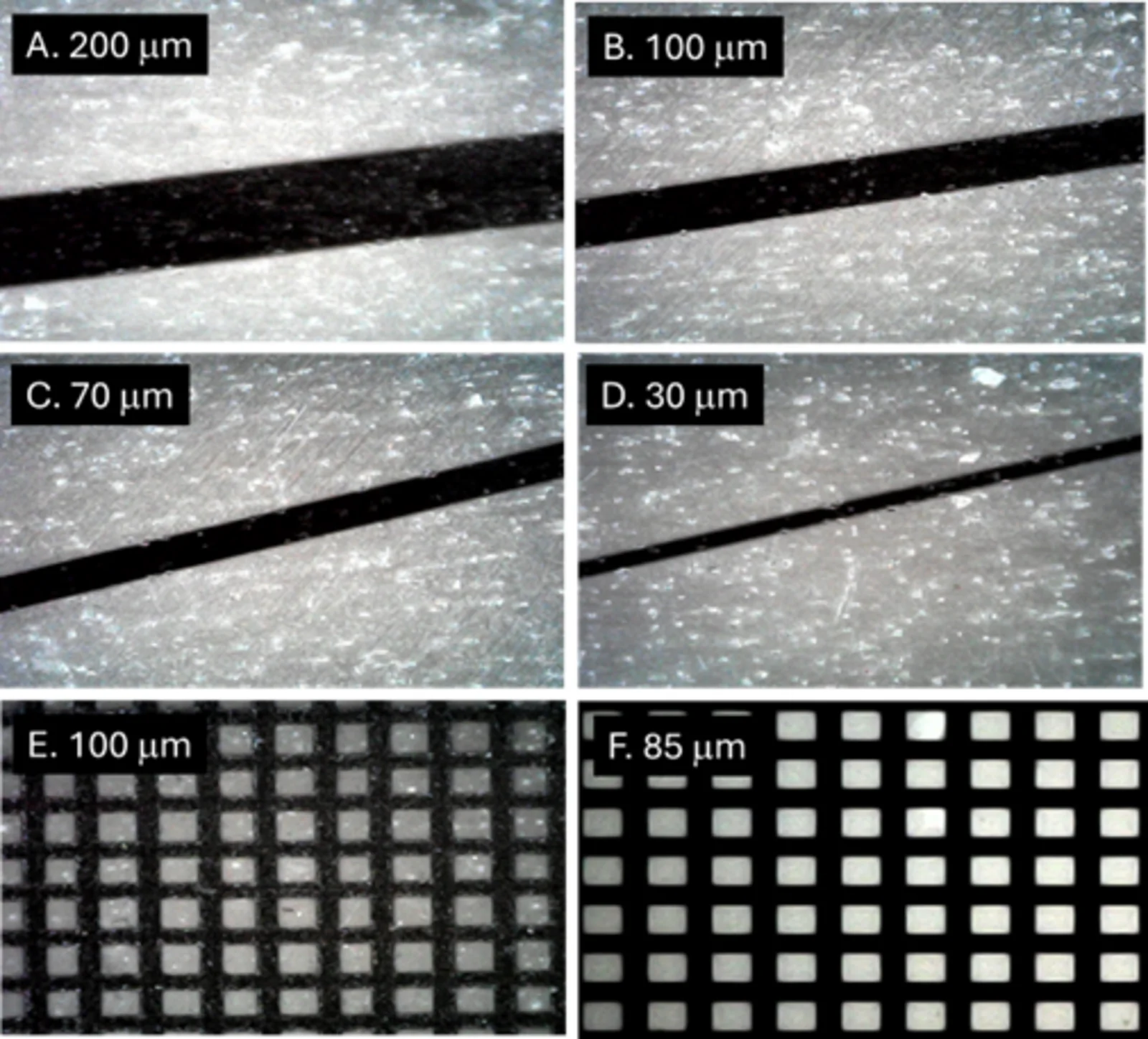
Calibrating the wired DMD. (A–D) Plastic calibration ruler with black lines of defined thickness ranging from 30 to 200 µm. (E) Plastic calibration ruler with 100 µm × 100 µm grid. (F) Electron microscope 300-mesh grid with a pitch of 85 µm (31 µm bar width, 54 µm hole width).

**Figure 4:**
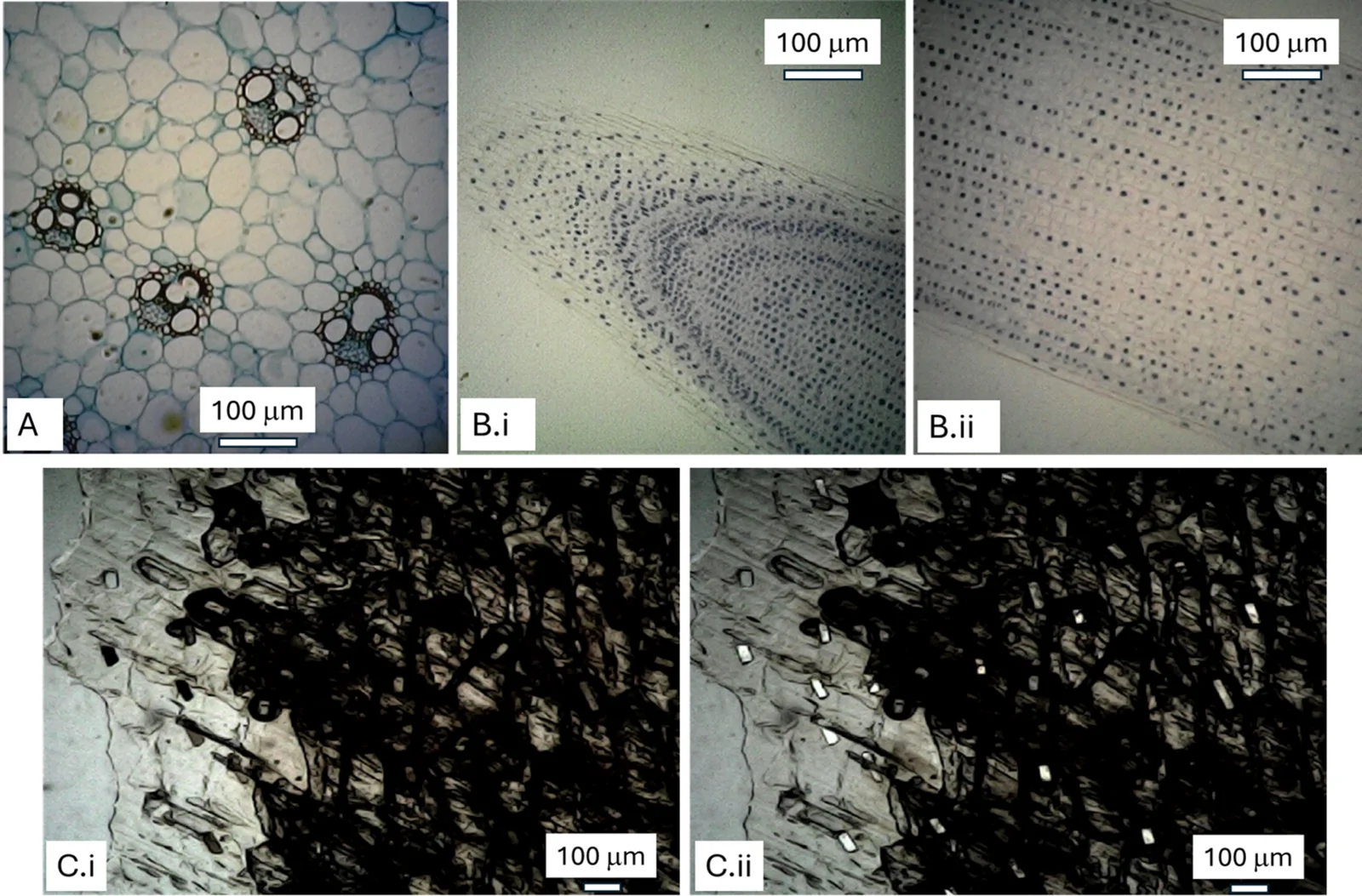
Plant tissues imaged using the Wi-Fi DMD. (A) Cross-section of a corn stem in a prepared slide. (B) Longitudinal section of an onion root tip, stained to highlight cell nuclei (dark staining objects) in a prepared slide. (B.i) Root tip. (B.ii) A view of the root at some distance from the tip. Some cells contain two closely positioned dark-staining structures, consistent with mitotic activity in this rapidly growing tissue. (C) Red onion tunic. (C.i) Unpolarized light. (C.ii) Polarized light, revealing birefringent calcium oxalate crystals as bright objects in or on tunic cells.

**Figure 5:**
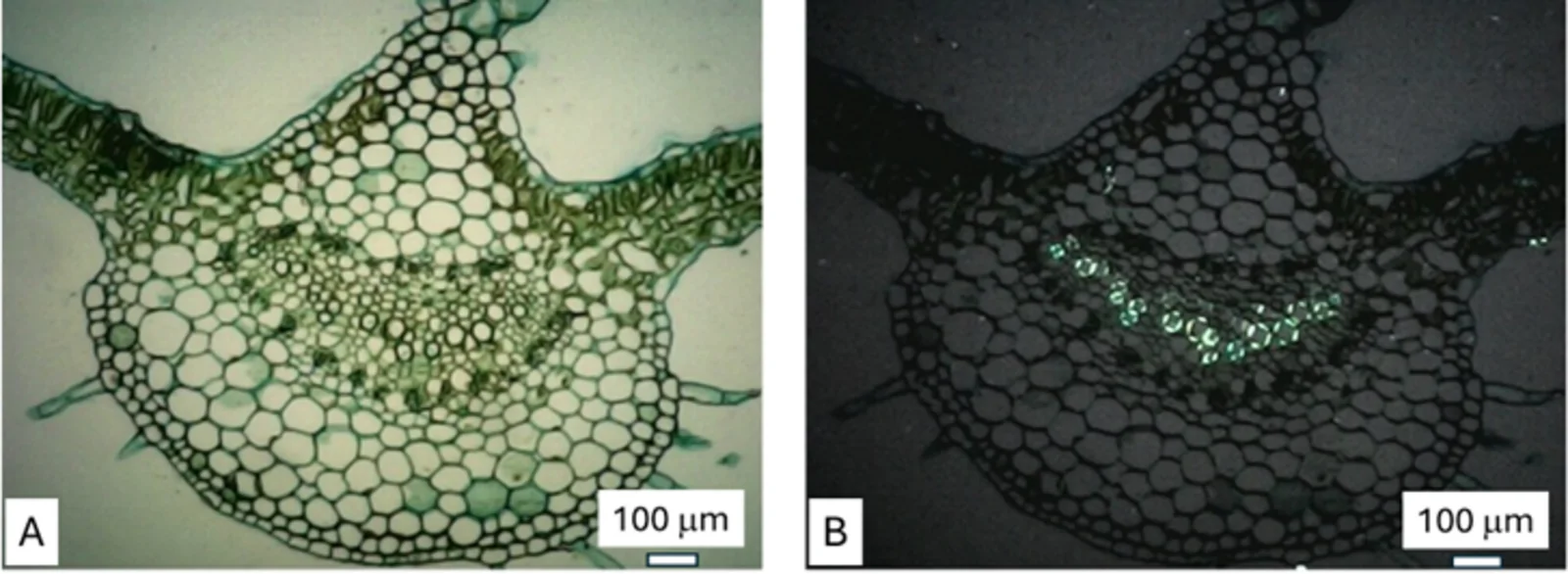
Cross-section of a tomato leaf in a prepared slide imaged with different degrees of polarized light using the Wi-Fi DMD. (A) Unpolarized. (B) Almost completely polarized, revealing birefringent material (bright white) in the vascular bundle region of the leaf’s midrib.

**Figure 6:**
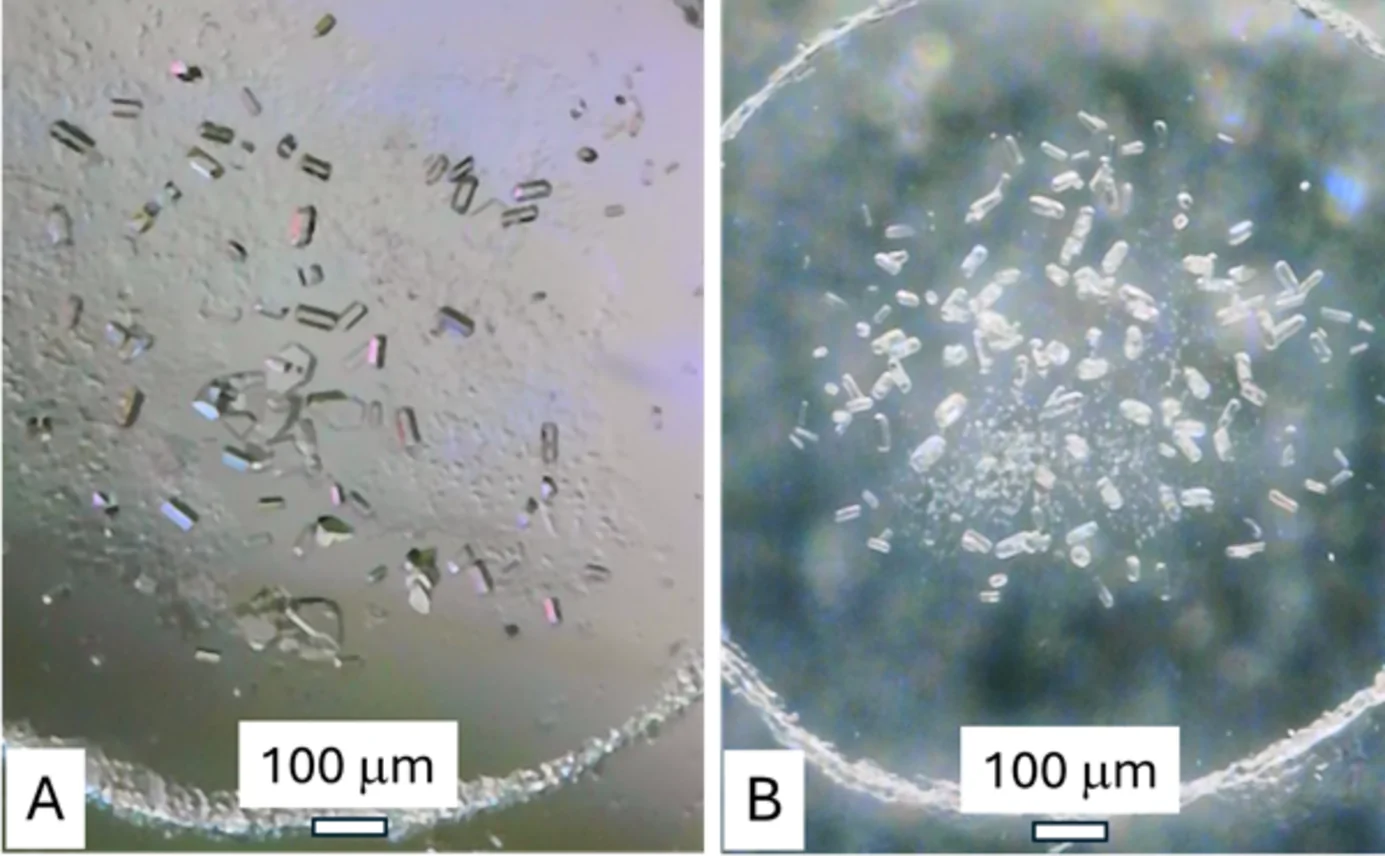
Lysozyme crystals suspended in the viscous lipid cubic phase surrounded by precipitant in glass sandwich plates at RT imaged with the wired DMD. (A) 5 hour-old crystals. (B) 3 day-old crystals.

**Figure 7:**
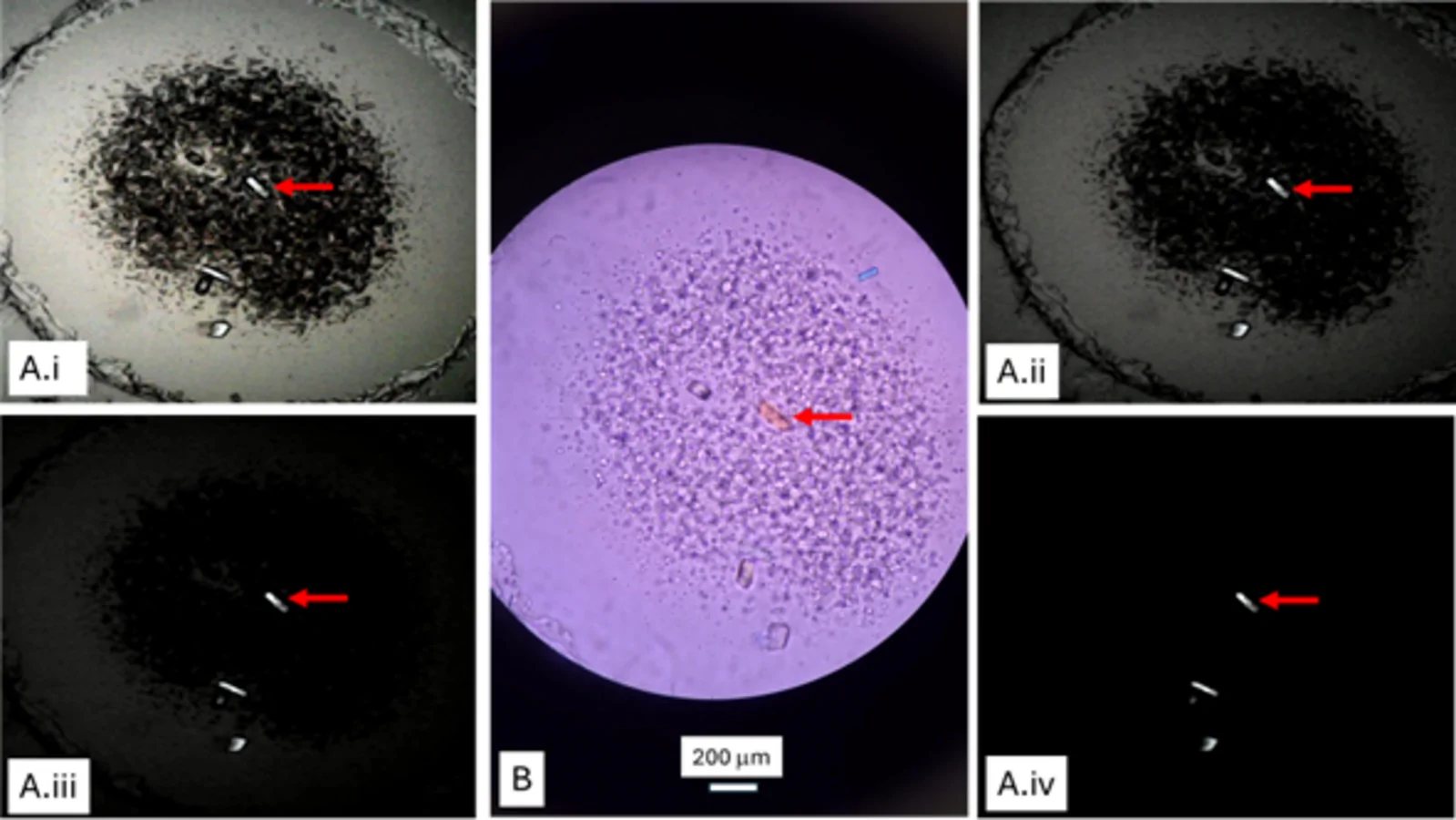
Lysozyme crystals in the LCP where droplets are present in the mesophase. Droplets can obscure the crystals when viewed with unpolarized light. The crystals light up and become more visible when polarized light is used. (A.i–A.iv) Images recorded with the polarizers progressively approaching the fully crossed condition with the wired DMD. (B) Image recorded with a Nikon microscope (10× ocular and 10× objective lenses) with polarizers partially crossed. Images were recorded when crystals were 1 day old. The crystal at the arrow is ∼80 µm long.

**Figure 8:**
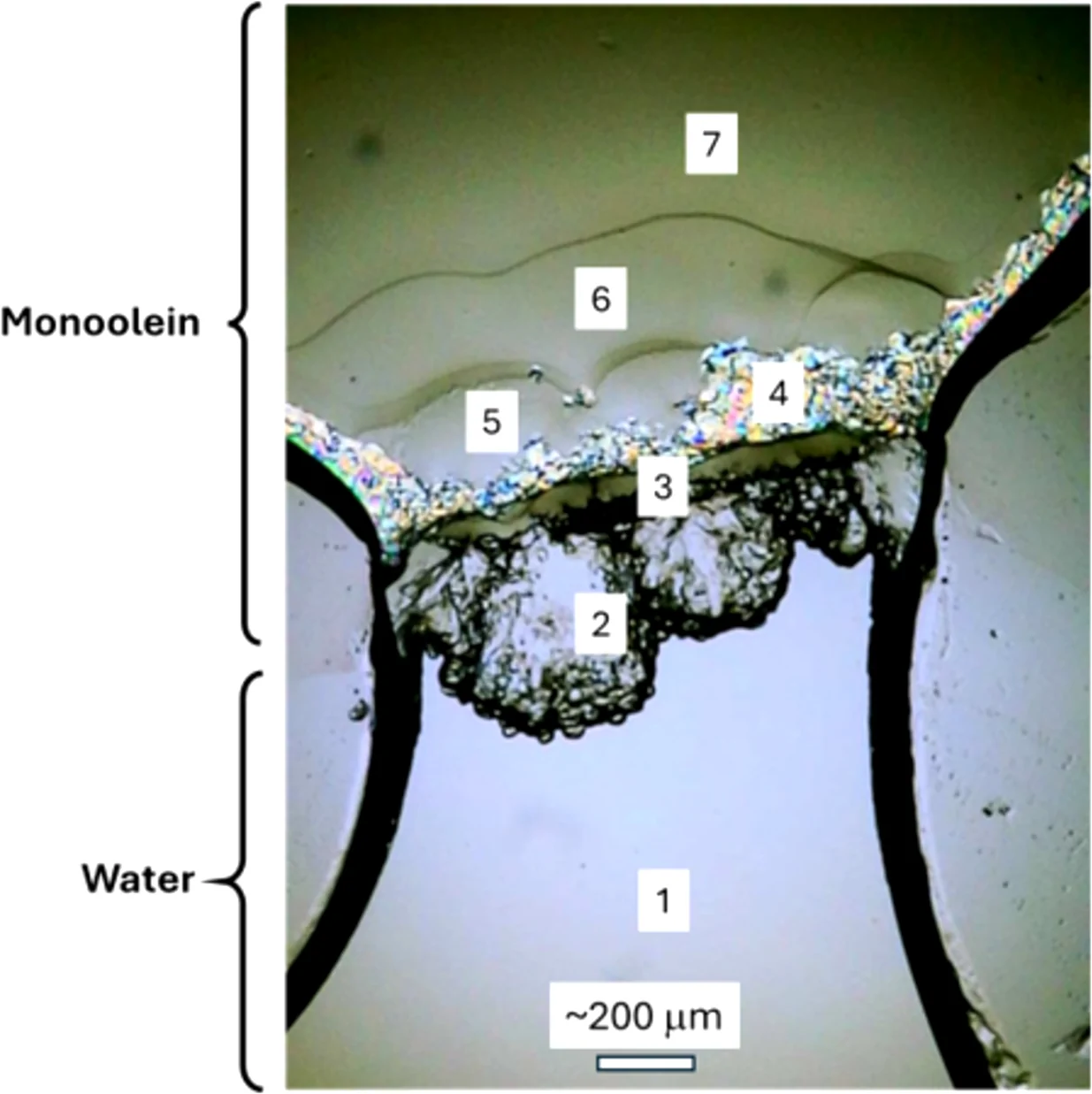
Image recorded with the Wi-Fi DMD at the monoolein/water interface at RT after 1 h. The sequence of phases expected on the basis of the temperature–composition phase diagram of the monoolein/water system (Qiu & Caffrey, 2000 ▸) includes (1) liquid water, (2) cubic *Pn*3*m* phase, (3) cubic *Ia*3*d* phase, (4) lamellar phase (birefringent) and (5–7) fluid isotropic phase variants.

**Figure 9:**
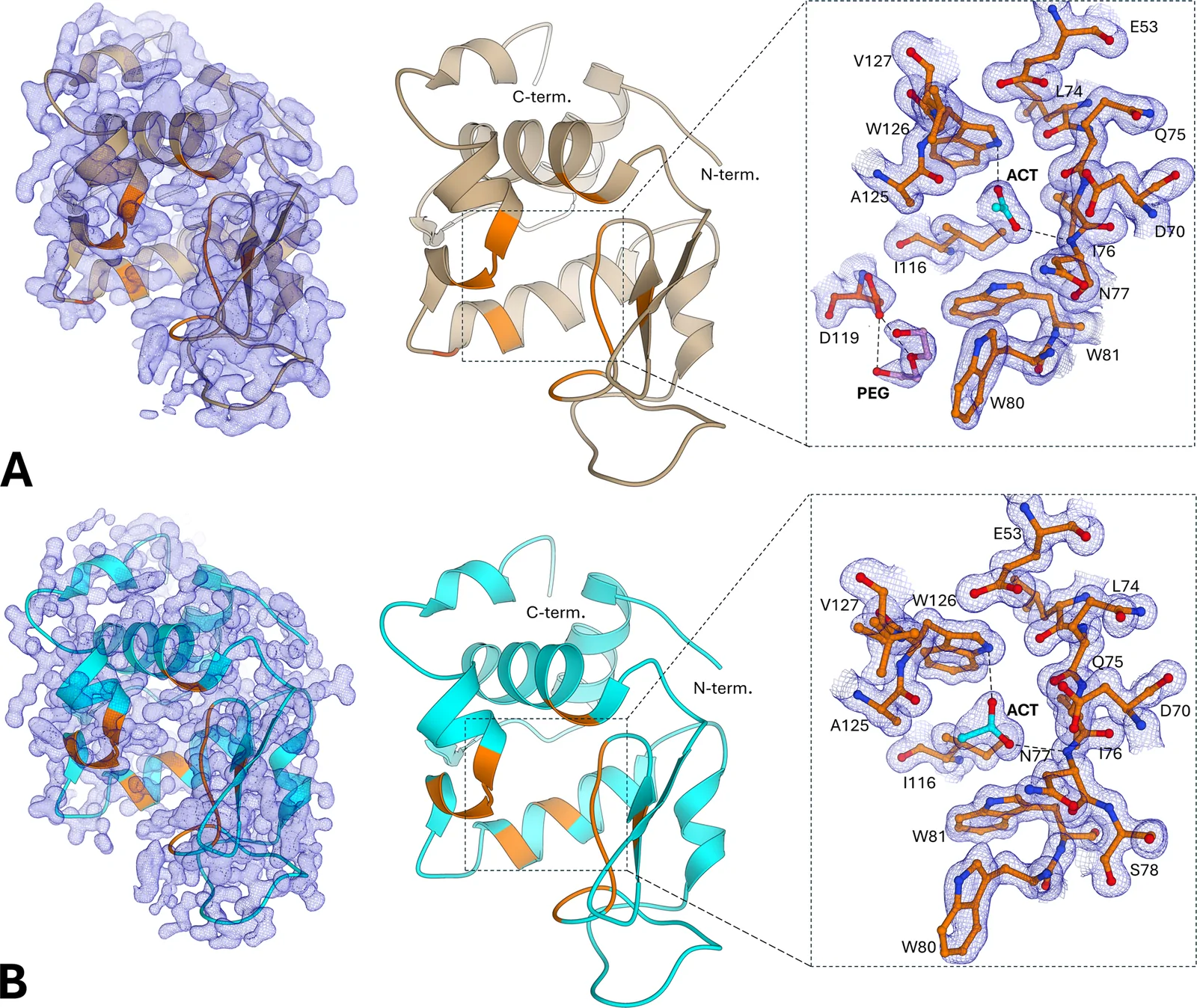
Lysozyme structures solved using crystals harvested from vapour-diffusion and glass sandwich plates with the assistance of the Wi-Fi DMD. (A) Structure from *in meso*-grown crystals at 1.74 Å resolution. (B) Structure from hanging-drop-grown crystals at 1.07 Å resolution. Left panels: models in cartoon representation with 2Fo–Fc electron density map contoured at 1.5σ. Middle panels: models in cartoon representation. Right panels: stick models of residues in the vicinity of the active site region marked by an acetate molecule (cyan carbons) from the precipitant solution. Electron density map contoured at 1.5σ. Figures were created in *Chimera 1.18* (Pettersen *et al.*, 2004 ▸).

**Figure 10:**
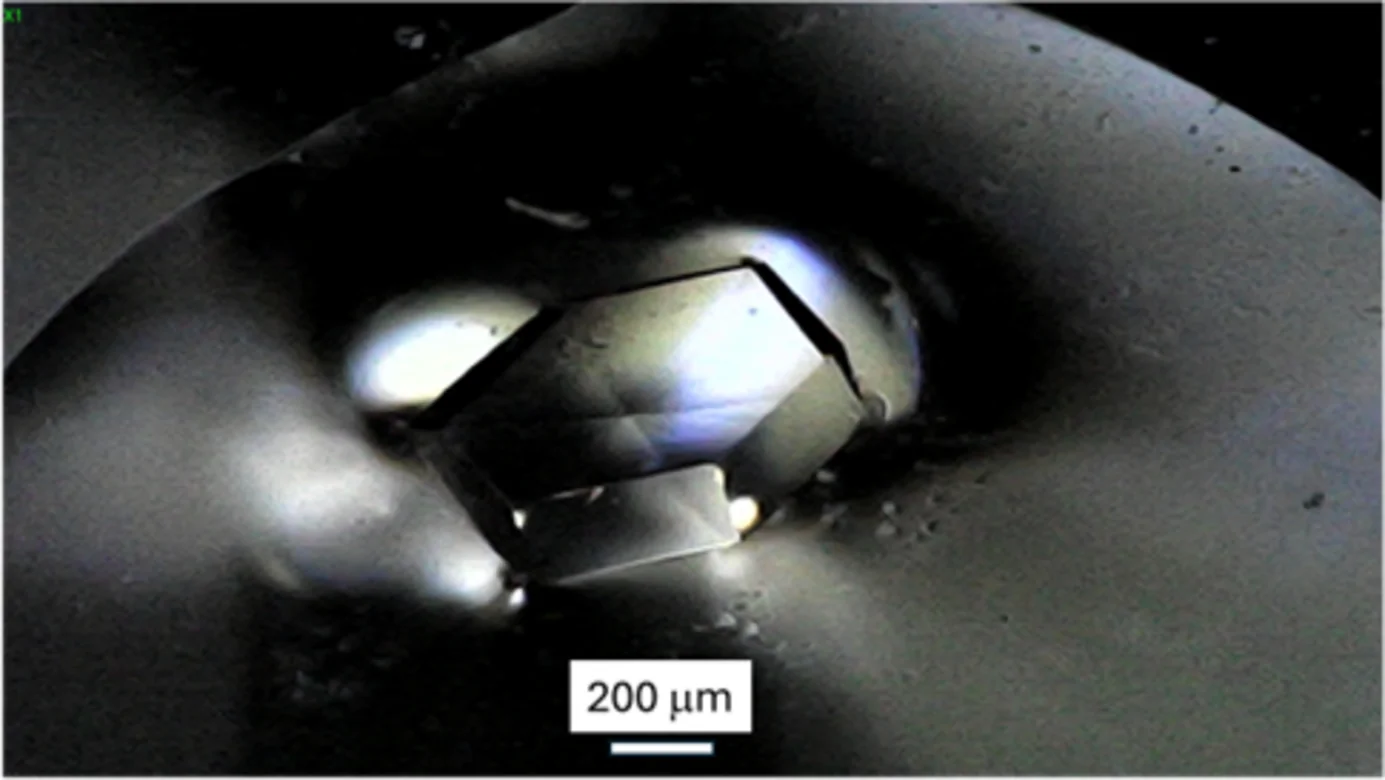
Lysozyme crystal in a 24-well hanging-drop crystallization plate at RT imaged with the wired DMD with top lighting only.

**Figure 11:**
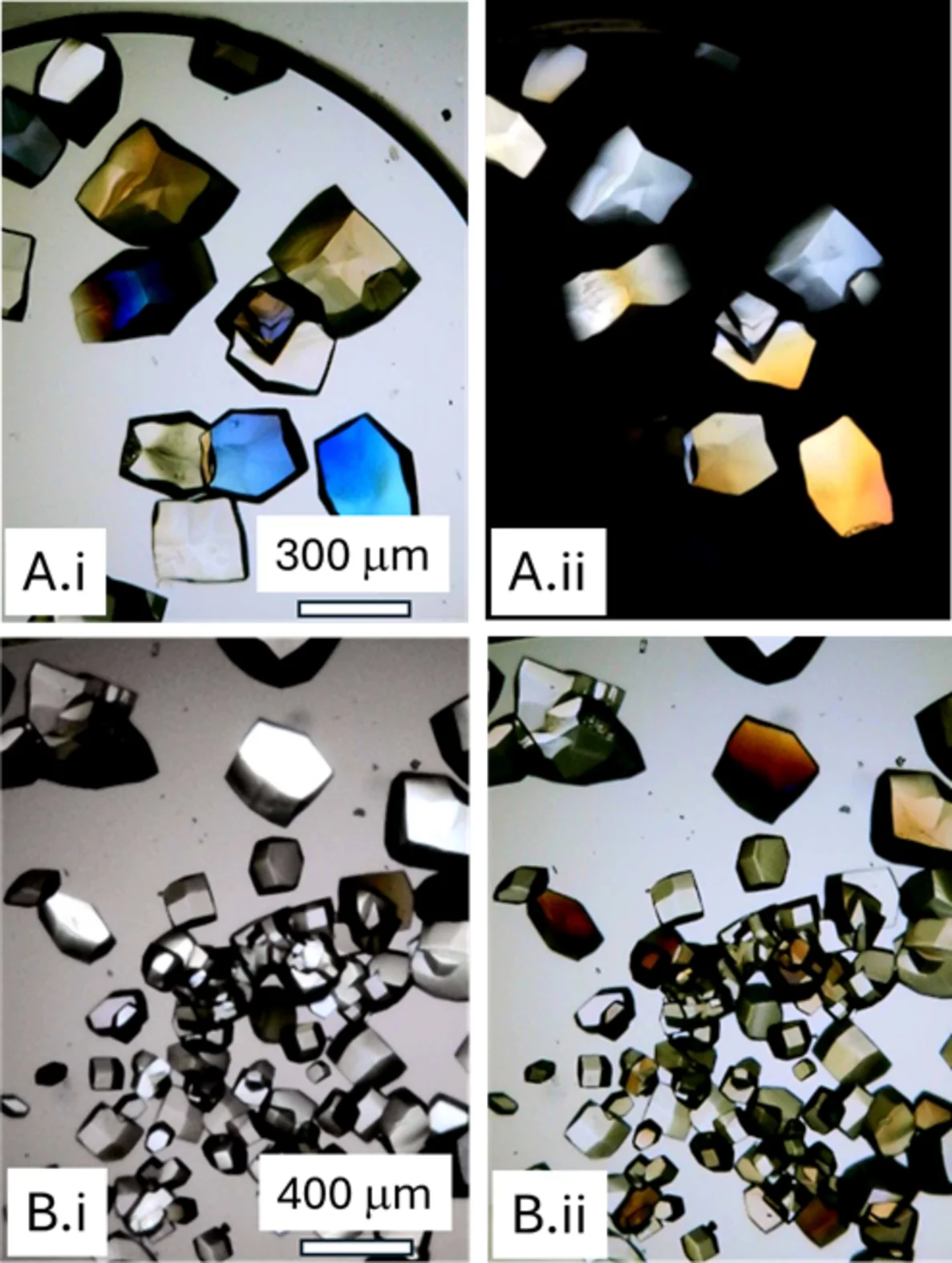
Lysozyme crystals grown in batch on glass sandwich plates imaged with the Wi-Fi DMD. (A.i) Polarizers partially crossed. (A.ii) Polarizers fully crossed. (B.i) Polarizers parallel (not crossed). (B.ii) Polarizers partially crossed.

**Table 1:** X-ray diffraction data processing and refinement statistics for hanging-drop- and *in meso*-grown lysozyme crystals

Crystal type	Hanging drop	*In meso*
Data processing statistics		
PDB ID	30da	30db
Beamline	DLS I04	DLS I04
Wavelength (Å)	0.95373	0.95373
Temperature (K)	100	100
Space group	*P*4_3_2_1_2	*P*4_3_2_1_2
Unit-cell dimensions		
*a* = *b*, *c* (Å)	78.5, 37.3	77.3, 38.5
α = β = γ (°)	90.0	90.0
Data range (°)	360	100
Oscillation (°)	0.1	0.1
Resolution range†	80–1.07 (1.14–1.07)	40–1.74 (1.85–1.74)
Reflections observed/unique	1127001/51194	88660/12471
Multiplicity	22.01	7.1
Completeness (%)	99.1 (94.3)	99.4 (98.6)
*R*_meas_ (%)	8.8 (754.4)	16.1 (274.2)
*R*_merge_ (%)	8.6 (710.6)	14.9 (254.8)
*I*/σ(*I*)	19.25 (0.88)	9.98 (1.17)
CC_1/2_	99.9 (42.7)	99.7 (47.9)

Refinement statistics		
Unique/test reflections	51002/1998	12467/998
*R*_work_	0.15	0.177
*R*_free_	0.173	0.206
Protein chains in asymmetric unit	1	1
Matthews coefficient (Å^3^ Da^−1^)	2.01	2.01
Solvent (%)	38.78	38.83
ADP model	Individual anisotropic	TLS + individual isotropic
Wilson *B* factor	13.0	23.6
〈*B*〉 for protein/ligands/water (Å^2^)	14.7/25.5/28.1	24.3/42.3/31.5
Non-hydrogen protein/ligand/solvent atoms	1065/12/191	1001/28/120
RMS bonds (Å)/angles (°)	0.008/0.99	0.015/0.81
Ramachandran plot (%) favoured/allowed/outliers	99.2/0.8/0	98.4/1.6/0

† Values in parentheses correspond to the highest-resolution shell.

## Data Availability

Data supporting the results can be obtained from the authors upon reasonable request. Structures have been deposited in the Protein Data Bank under accession codes 30da (hanging-drop structure, 1.07 Å) and 30db (*in meso* structure, 1.74 Å).
