# Supplementary material for: A low-cost, portable polarizing digital light microscope for monitoring crystal growth and for crystal harvesting
Source: J Appl Crystallogr. 2026 Sep 4;59(Pt 5):1693–706. doi: 10.1107/S1600576726007880 (PMC13636041; doi:10.1107/S1600576726007880)
Supplement: Supplementary file 1 [file j-59-01693-sup1.pdf]

JOURNAL OF APPLIED CRYSTALLOGRAPHY

Volume 59 (2026)

Supporting information for article:

A low-cost, portable polarizing digital light microscope for monitoring crystal growth and for crystal harvesting

Sarah Kennedy, Ryan H. Huang, Wojciech Witek, Pawel Krawinski, Coilín Boland, Linjie Yang, Neal Leddy and Martin Caffrey

Supporting information

Digital Microscope Device Parts List

Digital microscopes with plastic calibration ruler. Wired. Shenzhen Bobby E-Commerce Co., Ltd. App: HiView. Wi-Fi: W05A-WiFi Digital Microscope. Mafiti. App: DLscope.

Velmex Unislide translation slide (https://velmex.com/product-category/unislide/a15-series).

Polylactic acid. Elegoo.

Light emitting diode (AC/DC 12 ~ 18V SMD 1-LED). RUNCCI-YUN.

Polarizing filter (50 x 50 mm Linear Polarizing Film (XP42-18)). Edmund Optics.

Battery (CR2032). Panasonic.

Battery holder (CR2032 Battery Holder 1 or 2 x 3 V Button Battery Holder Coin Cell Battery Socket Holder Case Box with Wire Lead ON-OFF). RUNCCI-YUN.

Dimmer switch (DC 12v PWM motor speed controller, 1.8V, 3V, 5V, 6V, 12 V, 2A Speed Adjustable Sitch Module, Variable Voltage Regulator Dimmer Governor Switching Build with LED Indicator and Switch Function). RUNCCI-YUN.

SuperGlue. Locktite.

Hot glue gun, glue stick. The Art & Hobby Shop.

Crystallographic Information Files (CIF)

CIFs for the two PDB records are combined in the one CIF.

STL Files

For images of the components in the following STL files, see **Fig. S3**.

Disc

DM Holder

LED Support Rod

Lid

Paddle for Polarizing Filter

Spacer

Tray

Figures

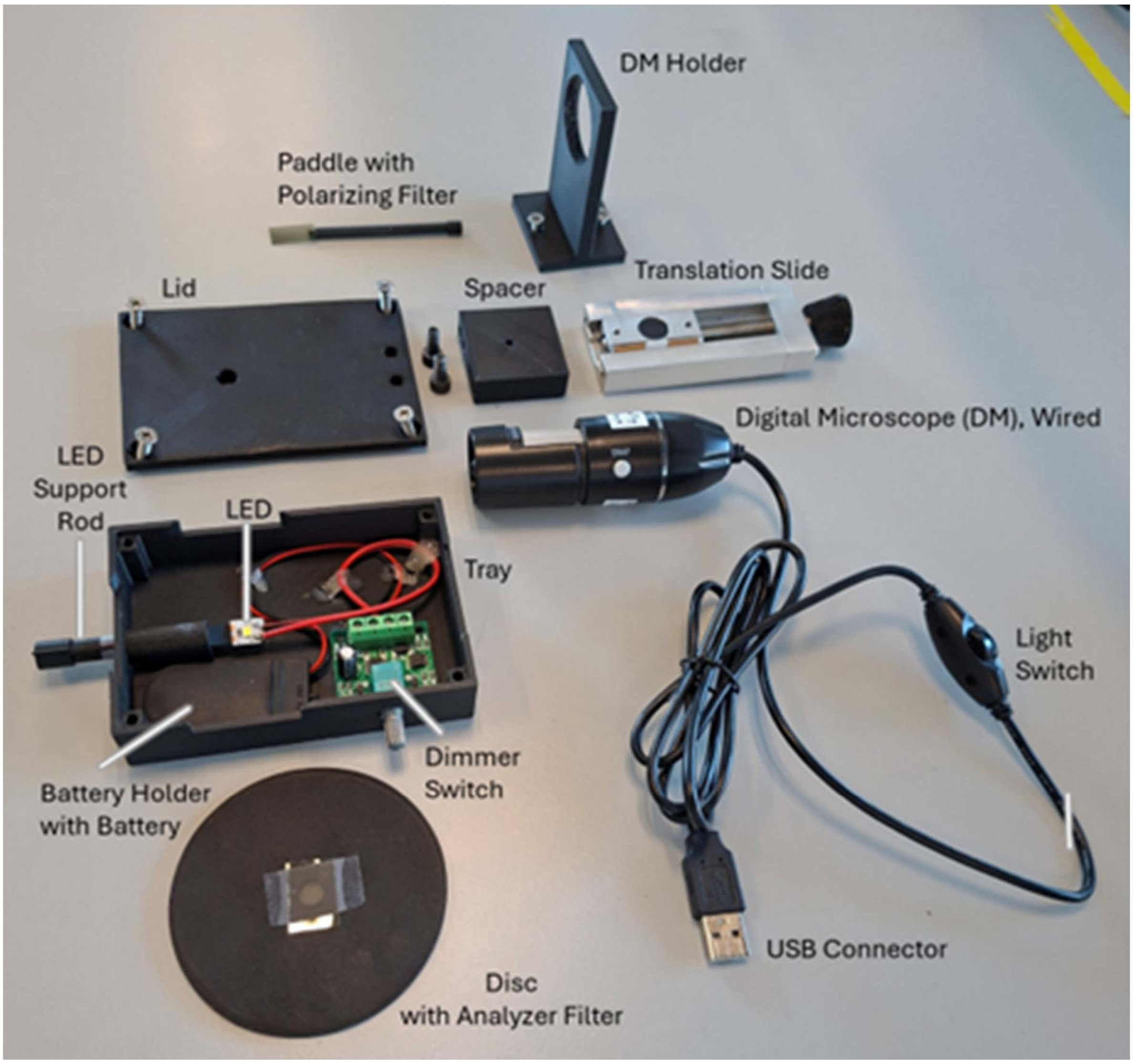

Fig. S1. Parts of the Digital Microscope Device. The wired DMD is shown here.

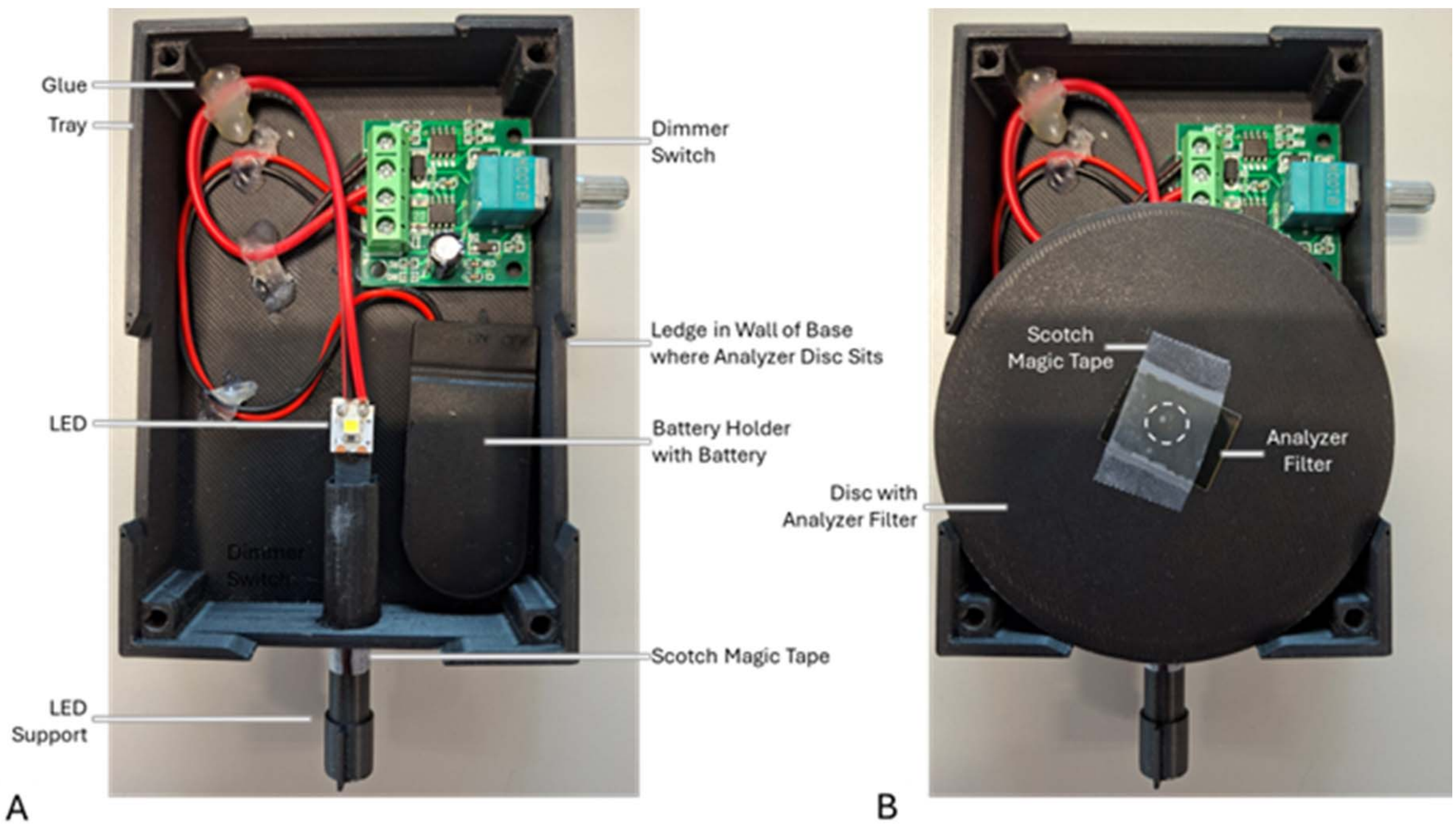

Fig. S2. View inside the tray of the Digital Microscope Device. (A) Without the analyzer disc in place. (B) With the analyzer disc in place. The dashed circle demarks the aperture in the disc positioned above the LED that is masked by the analyzer filter.

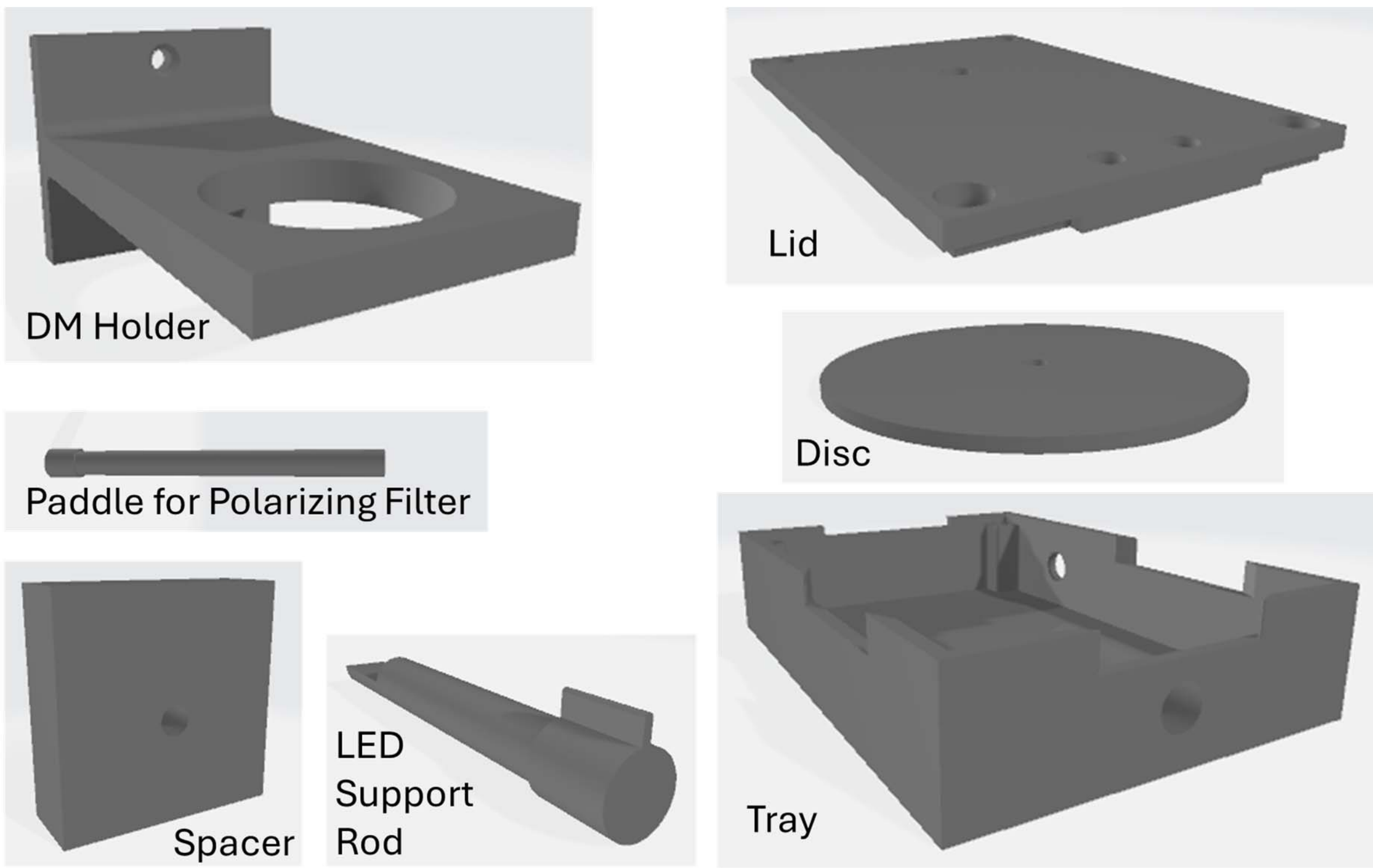

Fig. S3. 3D printed components of the Digital Microscope Device for which STL files are available under Supplementary Information.

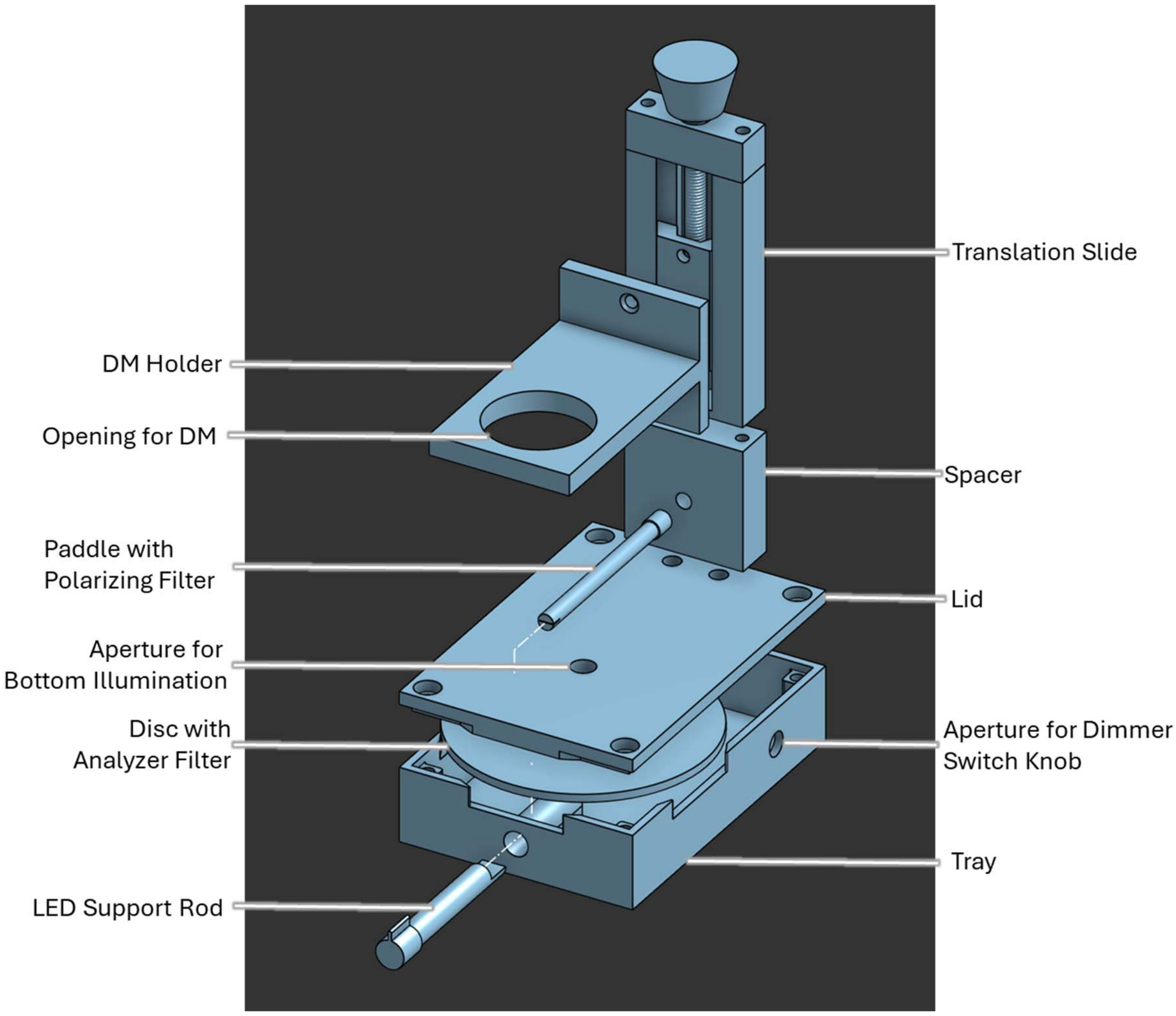

Fig. S4. Perspective drawing of the Digital Microscope Device parts shown in relation to one another. The components that reside inside the base, that include the battery housing and battery, dimmer switch, and LED, are omitted for clarity.

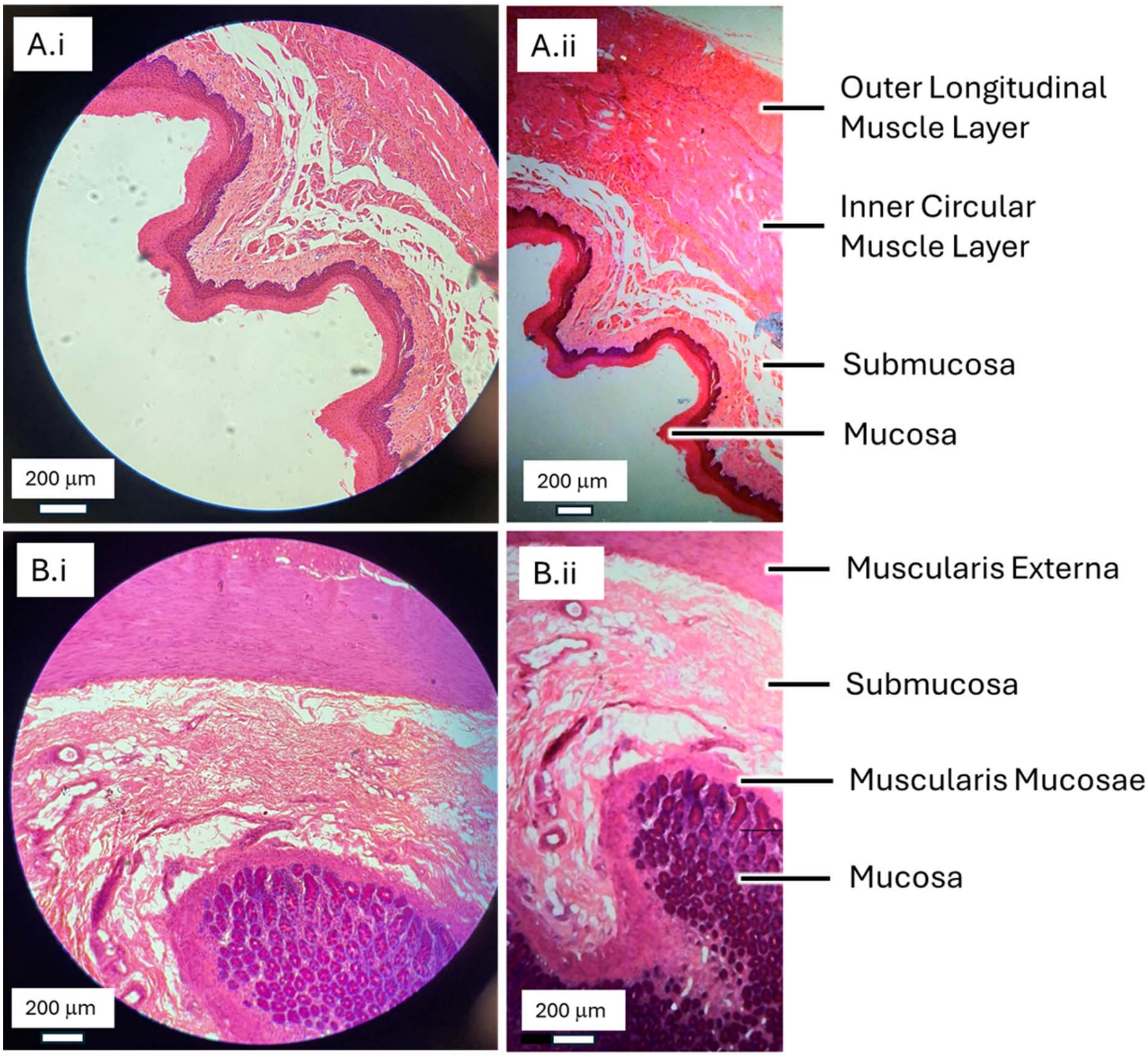

Fig. S5. Comparison of oesophagus and colon histology visualised using a standard laboratory benchtop microscope versus the prototype Wi-Fi DMD polarising microscope. (A.i) Transverse section of oesophagus (H&E stain), photographed through a standard laboratory benchtop microscope using an iPhone 12. (A.ii) The same oesophagus section visualised and photographed using the Wi-Fi DMD prototype, with tissue layers annotated. The outer longitudinal muscle layer and the inner circular muscle layer together comprise the muscularis externa. (B.i) Transverse section of colon (H&E stain), photographed through a standard laboratory benchtop microscope using an iPhone 12. (B.ii) The same colon section visualised and photographed using the Wi-Fi DMD prototype, with tissue layers annotated. A photograph of the glass slides used for panels A and B is shown in Fig. S6.

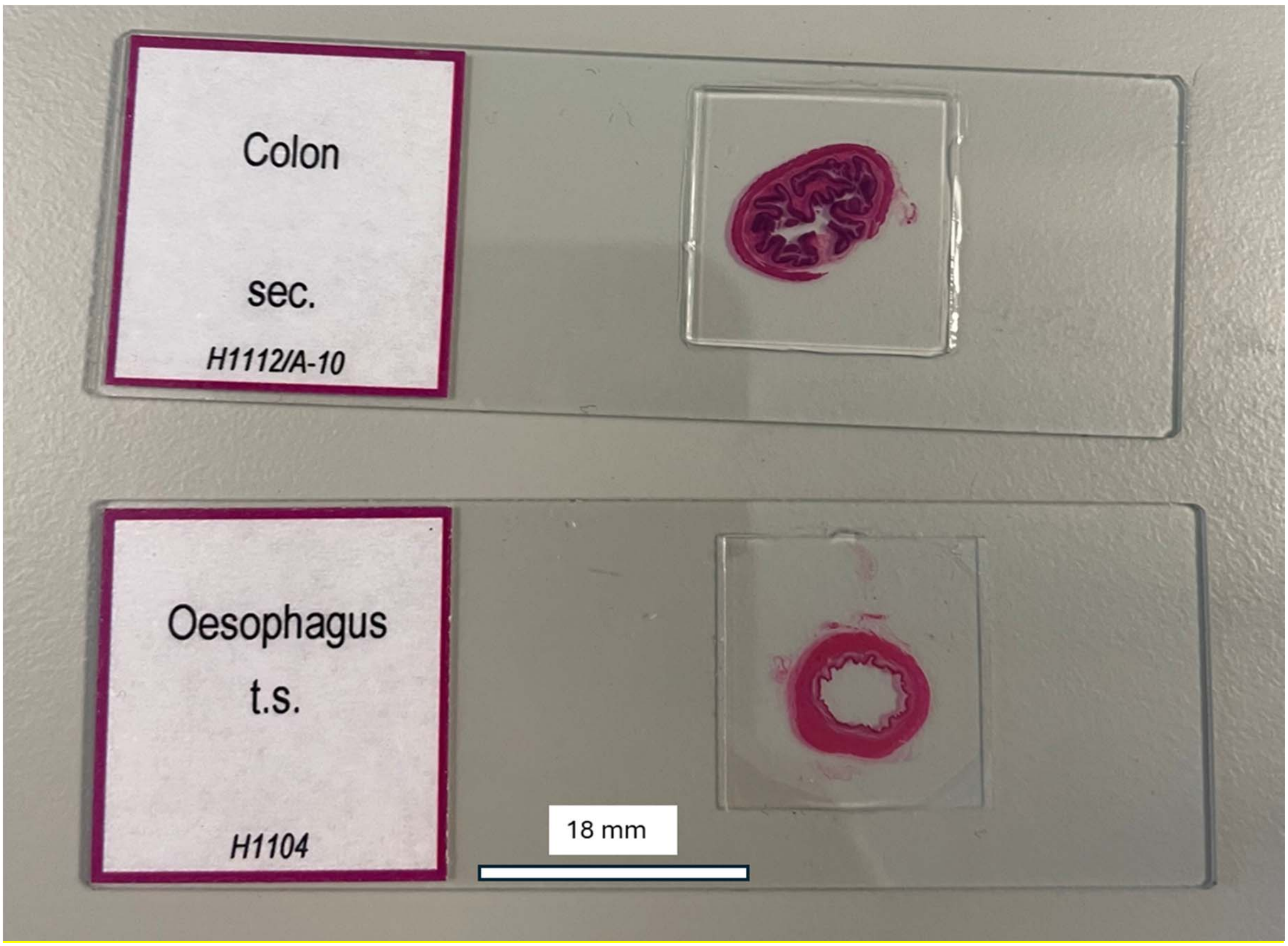

Fig. S6. Photograph of the histology slides used for panels A and B in Fig. S5. Specimen codes H1112/A-10 [colon] and H1104 [oesophagus]). The species of origin was not confirmed by the laboratory supplying the histology slides; specimens are presumed rodent based on tissue morphology and scale.

Movies

Movie S1. Growth of batch lysozyme crystals in glass sandwich plates at RT recorded over a 6 h period with the Wi-Fi DMD and a cell phone. Images were annotated with an elapsed time stamp and a scale bar.

Movie S2. Growth of batch lysozyme crystals in glass sandwich plates for ~10 min in a cold cabinet at 4 °C followed by a 6 h incubation at RT. Images were recorded remotely at RT with the Wi-Fi DMD and a cell phone. Images were annotated with an elapsed time stamp and a scale bar.

Acknowledgements

We thank Daniel Trimble and Diarmuid Balfe (Department of Mechanical and Manufacturing Engineering, Trinity College Dublin) for assistance with 3D printing, Sarah-Louise Hassett (Physiology Department, Trinity College Dublin) for advice on Fig. S5 and Fig. S6, and the Diamond Light Source synchrotron facility for X-ray beam time allocations and the staff at beamline I04 for help with data collection. The past and present members of the Membrane Structural and Functional Biology group are acknowledged for their assorted contributions to this study.

Conflict of Interest

There are no conflicts of interest.

Data availability

Data supporting the results can be obtained from the authors upon reasonable request. Structures have been deposited in the Protein Data Bank under accession codes: 30DA (Hanging drop structure, 1.07 Å); 30DB (In meso structure, 1.74 Å).

Funding information

This work was supported by Research Ireland (Grant No. 22/FFP-A/10278) and The Biochemical Society (Grant No. DIS-433).

References

Aherne, M., Lyons, J. A. & Caffrey, M. (2012). *J. Appl. Cryst.* **45**, 1330-1333.

Afonine, P. V., R. Grosse-Kunstleve, W., Echols, N., Headd, J. J., Moriarty, N. W., Mustyakimov, M., Terwilliger, T. C., Urzhumtsev, A., Zwart, P. H. & Adams, P. D. (2012). *Acta Cryst.* D**68**: 352-367.

Bergfors, T. M. (2021). (ed.) Protein Crystallization. 3rd Edition (Ebook), IUL Biotechnology Series, 10.

Caffrey, M. Chapter 17 in Bergfors, T. M. (2021). (ed.) Protein Crystallization. 3rd Edition (Ebook), IUL Biotechnology Series, 10.

Caffrey, M. & Cherezov, V. (2009)**.** *Nat. Protoc*. **4**, 706-731.

Caffrey, M. & Porter, C. (2010). *J. Vis. Exp.* **45**, e1712. doi: 10.3791/1712.

Cheng, A., Hummel, B., Qiu, H. & Caffrey, M. (1998). *Chem. Phys. Lipids*. **95**, 11-21.

Emsley, P. & Cowtan, K. (2004). *Acta Cryst.* D**60**: 2126-2132.

Kabsch, W. (2010). *Acta Cryst.* D**66**: 125-132.

Kowiel, M., Jaskolski, M. & Dauter, Z. (2014). *Acta Cryst.* D**70**: 3290-3298.

Li, D., Boland, C., Aragão, D., Walsh, K. & Caffrey, M. (2012). *J. Vis. Exp.* **67**, e4001.

McCoy, A. J., Grosse-Kunstleve, R. W., Adams, P. D., Winn, M. D., Storoni, L. C., & Read, R. J.(2007). *J. Appl. Cryst.* **40**, 658-674.

McPherson, A. (1999). Crystallization of biological macromolecules. Cold Spring Harbor Laboratory Press. Cold Spring Harbor, NY.

Pettersen, E. F., Goddard, T. D., Huang, C. C., Couch, G. S., Greenblatt, D. M., Meng, E. C. & Ferrin, T. E. (2004) *J. Comput. Chem*. **25**, 1605-1612.

Qiu, H. & Caffrey, M. (2000). *Biomaterials*. **21**, 223-234.

Rupp, B. (2010). *Biomolecular Crystallography: Principles, Practice, and Application to Structural Biology*. Garland Science, New York, NY.

Tarver, C. L. & Pusey, M. (2017). *Acta Cryst.* F**73**, 657-663.
